# New Zinc-Based Active Chitosan Films: Physicochemical Characterization, Antioxidant, and Antimicrobial Properties

**DOI:** 10.3389/fchem.2022.884059

**Published:** 2022-05-31

**Authors:** Debora Policastro, Eugenia Giorno, Francesca Scarpelli, Nicolas Godbert, Loredana Ricciardi, Alessandra Crispini, Angela Candreva, Fabio Marchetti, Sonila Xhafa, Renata De Rose, Antonello Nucera, Riccardo C. Barberi, Marco Castriota, Loredana De Bartolo, Iolinda Aiello

**Affiliations:** ^1^ MAT-INLAB (Laboratorio di Materiali Molecolari Inorganici) and LASCAMM - CR INSTM, Unità INSTM of Calabria, Department of Chemistry and Chemical Technologies, University of Calabria Ponte Bucci, Rende, Italy; ^2^ CNR NANOTEC- Institute of Nanotechnology U.O.S. Cosenza, Rende, Italy; ^3^ School of Science and Technology Chemistry Section, University of Camerino, Macerata, Italy; ^4^ LAB CF-INABEC Department of Chemistry and Chemical Technologies, University of Calabria, Rende, Italy; ^5^ Department of Physics, University of Calabria Ponte Bucci, Rende, Italy; ^6^ Institute on Membrane Technology, National Research Council of Italy, C/o University of Calabria, Rende, Italy

**Keywords:** chitosan, Zn(II) complex, films, antioxidant activity, antimicrobial activity

## Abstract

The improvement of the antioxidant and antimicrobial activities of chitosan (CS) films can be realized by incorporating transition metal complexes as active components. In this context, bioactive films were prepared by embedding a newly synthesized acylpyrazolonate Zn(II) complex, [Zn(Q^PhtBu^)_2_(MeOH)_2_], into the eco-friendly biopolymer CS matrix. Homogeneous, amorphous, flexible, and transparent CS@Zn_n_ films were obtained through the solvent casting method in dilute acidic solution, using different weight ratios of the Zn(II) complex to CS and characterized by powder X-ray diffraction (PXRD), thermogravimetric analysis (TGA), differential scanning calorimetry (DSC), Fourier transform infrared (FT-IR), Raman, and scanning electron microscopy (SEM) techniques. The X-ray single-crystal analysis of [Zn(Q^PhtBu^)_2_(MeOH)_2_] and the evaluation of its intermolecular interactions with a protonated glucosamine fragment through hydrogen bond propensity (HBP) calculations are reported. The effects of the different contents of the [Zn(Q^PhtBu^)_2_(MeOH)_2_] complex on the CS biological proprieties have been evaluated, proving that the new CS@Zn_n_ films show an improved antioxidant activity, tested according to the DPPH method, with respect to pure CS, related to the concentration of the incorporated Zn(II) complex. Finally, the CS@Zn_n_ films were tried out as antimicrobial agents, showing an increase in antimicrobial activity against Gram-positive bacteria (*Staphylococcus aureus*) with respect to pure CS, when detected by the agar disk-diffusion method.

## Introduction

Biodegradable polymers derived from renewable resources are considered nowadays as the future generation of coating materials for food packaging as well as biomedical devices (Vendra et al., 2011; Wasilewska and Winnicka 2019; Haghighi et al., 2020; Yewale et al., 2021). Although for biomedical applications, polymeric-based materials are still widely used for implants, drug delivery, filtration, and patches, they are often subject to rejection and incompatibility with the human organism (Vendra et al., 2011; Wasilewska and Winnicka 2019). Among the most abundant biopolymers in nature, chitosan (CS) is an emerging and promising material used in several areas, such as agriculture, cosmetology, pharmaceutical, and medical devices, due to its non-toxicity, biocompatibility, biodegradability, and bioactivity (Darmadji and Izumimoto 1994; Ravi Kumar 2000; Kumar et al., 2004; Dutta et al., 2009; Aranaz et al., 2018; Afonso et al., 2019; Jiménez-Gómez and Cecilia 2020; Díaz-Montes and Castro-Muñoz 2021). CS is a linear polysaccharide of (1,4)-linked 2-amino-deoxy-β-d-glucose derived by the deacetylation of chitin, the major component of the shell of crustacea, such as crawfish, shrimp, and crab (Ravi Kumar 2000; Jiménez-Gómez and Cecilia 2020). CS is insoluble in water and organic solvents but dissolves in dilute acid solutions acquiring positive charges through the protonation of the amine groups and behaving as a polycationic water-soluble polymer (Melro et al., 2021). Once dissolved, CS can easily give rise to different formulations spanning from various sized particles to gels, fibers, and films, exhibiting antioxidant, antimicrobial, and antifungal activities, favorably used for several biomedical applications (Zhang et al., 2009; Anitha et al., 2014; Afonso et al., 2019; Dongre and Longbiao 2019; Abd El-Hack et al., 2020; Li and Zhuang 2020). The film-forming properties of CS allow the preparation of films with good mechanical properties, selective gas permeability, and good entrapping properties for biologically active compounds. Indeed, chemical modification and/or mixing with other components (including plasticizers) can improve the functional properties of CS films, receiving particular attention as a bioabsorbable polymer with drug delivery capability in its film formulation when added with pharmacologically active molecules and vaccine adjuvant (Vieira et al., 2011; Pokhrel and Yadav 2019; Rathore et al., 2019; Idris et al., 2021). Due to the presence of positive charges at physiological pH, arising from protonation of many NH_2_-groups, CS films may bind to negatively charged surfaces like biological tissues, giving rise to bio-adhesive property, which, combined with biocompatibility and biodegradability, yields good polymeric supports for film manufacturing (Mengatto et al., 2012). Moreover, the choice of suitable additives with different degrees of hydrophobicity can modulate the high CS film hydrophilicity, improving the durability and stability in water conditions. CS films loaded with many different drugs have been studied as transdermal drug delivery systems, proving that CS is an efficient carrier for small- and large-molecular-weight therapeutically effective drug molecules across the skin (Nair 2019). Useful approaches toward the improvement of the antibacterial and antioxidant activities of the CS film are the incorporation of silver, gold, and ZnO nanoparticles into the polymeric matrix (Lu et al., 2017; Ryan et al., 2017; Arroyo et al., 2020), as well as its functionalization with both bioactive essential transition metal ions and their metal complexes. It has been shown that Zn(II) and Cu(II) metal ions can be chelated by the amino groups on the polymeric backbone of CS, and it is worth noting that the Zn–CS composite shows interesting antimicrobial activities (Wang et al., 2004; Rogina et al., 2019). The use of transition metal complexes as active components for the functionalization of polymeric materials could represent a novel strategy toward the production of materials specifically tailored for their application, taking advantage of the inherent flexibility of transition metal complexes (Ardean et al., 2021). Functionalization of the CS backbone has proved to be an effective way to covalently bound transition metal complexes. For example, the *in situ* reaction between the two aldehydes of a Zn(II) vanillin complex, [Zn (phen) (van)_2_], and the CS amino groups yields the chemical modification of the polymeric film, effective in the treatment of cutaneous wounds of diabetic rats through the release of the active Zn(II) metal complex (de Aragão Tavares et al., 2019). Zinc (Zn) is the second-most abundant metal after iron found in the body and is essential for several cellular processes such as cell growth and cell division (Coleman 1998; Ugarte and Osborne 2014). The accumulation of Zn in the human body does not produce toxic effects, while, on the contrary, a lack of this metal ion causes severe injuries on the immune system (Stefanidou et al., 2006). Within the development of new metal-based drugs, wide interest is recently turned to the use of Zn(II) coordination complexes, with low toxicity and low side effects, in medicinal therapeutics and biosensors (Drewry and Gunning 2011; Gonnella et al., 2022). Our studies, among others, have been focused on the antitumor activity of Zn(II) complexes containing N,N-chelating ligands and acylpyrazolones, promising systems to verify the metal–ligand synergistic effects (Liguori et al., 2010; Marchetti et al., 2020). Indeed, acylpyrazolone ligands are themselves biologically active, that the conjugation with their ability to chelate transition metal ions gives rise to metal complexes showing interesting features, such as anticancer, antioxidant, antifungal, and antimicrobial activities (Marchetti et al., 2015; Marchetti et al., 2019). In this scenario, we have synthesized and structurally characterized a new homoleptic acylpyrazolone-based Zn(II) complex, [Zn(Q^PhtBu^)_2_(MeOH)_2_], selected as a possible antioxidant and antimicrobial agent when incorporated into the CS polymer matrix using different weight ratios of the Zn(II) complex to CS. The new homogeneous, amorphous, flexible, and transparent composite films CS@(Zn)_n_ (*n* = 1.25, 2.5, 5, 7.5, 10% w/w), obtained through the solvent casting method in dilute acidic solution, are characterized by powder X-ray diffraction (PXRD), thermogravimetric analysis (TGA), differential scanning calorimetry (DSC), FT-IR, Raman, and SEM techniques. Investigation to rationalize the hydrogen-bonding interactions eventually formed between the [Zn(Q^PhtBu^)_2_(MeOH)_2_] complex and the CS backbone structure is conducted using X-ray single-crystal analysis and hydrogen bond propensity (HBP) calculations. The effects of the different contents of the [Zn(Q^PhtBu^)_2_(MeOH)_2_] complex on the antioxidant activity of the composite films CS@(Zn)_n_ are tested according to the DPPH method, as well as the antimicrobial activity detected against Gram-positive and Gram-negative bacteria (*Staphylococcus aureus* and *Escherichia coli*), by the agar disk-diffusion method.

## Experimental Section

### Materials

CS (molecular weight: 100,000–300,000 Da; degree of deacetylation: 90%) was purchased from Acros Organics (New Jersey, United States). The ligand HQ^PhtBu^ has been prepared according to the literature (Marchetti et al., 2000). All other chemicals were of analytical grade, purchased from Sigma-Aldrich (St. Louis, Missouri, United States), and were used without further purification.

### Characterization

Melting points were determined with a Leica DMLP polarizing microscope equipped with a Leica DFC280 camera and a CalCTec (Italy) heating stage. Elemental analyses were performed with a PerkinElmer 2400 CHNS/O analyzer. ^1^H and ^13^C NMR spectra were recorded on a 500Bruker Ascend (500 MHz for ^1^H, 125 MHz for ^13^C) instrument operating at room temperature relative to TMS. FT-IR spectroscopy spectra on the film samples were recorded using a Perkin Elmer Spectrum 100 FT-IR spectrometer in the mid-infrared area of 4000–450 cm^−1^. KBr pellets were used to only obtain the [Zn(Q^PhtBu^)_2_(MeOH)_2_] complex spectrum. Raman spectra were collected by a micro-Raman LABRAM apparatus by Horiba Jobin–Yvon Srl equipped by a 632.8 nm laser source of 17 mW power (He:Ne laser) and a ×50 objective by Olympus lens with a focal length of 15 mm. The spectral resolutions can be valued in 2 cm^−1^. TGA was performed on a Perkin Elmer Pyris 6 thermogravimetric analyzer. Approximately 3 mg of each film was placed in an alumina crucible and heated from 25 to 600°C, at a heating rate of 5°C min^−1^, under a dry nitrogen atmosphere. DSC measurements were carried out using a TA DSC Q2000 instrument with nitrogen as a purge gas, at a flow rate of 50 ml min^−1^. Accurately weighed samples (1.5–2 mg of each film) were sealed in non-hermetic aluminum pans and heated from 25 to 350°C with a heating rate of 10°C/min. Moreover, to check for reproducibility, measurements were repeated on three different samples taken from the same film. The film’s morphology was studied through SEM. Images of the films’ microstructure were acquired on a Phenom ProX desktop microscope (Thermo Fisher Scientific Inc., Waltham, MA, United States) equipped with a dedicated detector for energy-dispersive X-ray spectroscopy (EDX). Samples were deposited onto carbon-conductive, double-coated tabs and were observed in a backscattering mode at a 5 kV voltage without any additional conductive coating. Membranes’ sections were performed on samples that were cut under freezing conditions after immersion in a nitrogen liquid bath to obtain samples with clean and definite profiles.

### Preparation of [Zn(Q^PhtBu^)_2_(MeOH)_2_]

The synthesis of the [Zn(Q^PhtBu^)_2_(MeOH)_2_] was performed consistently with the existing literature (Liguori et al., 2010). Zn(CH_3_COO)_2_·2H_2_O (0.100 g, 0.455 mmol) was added to a solution of 30 ml of methanol containing 0.305 g (0.911 mmol) of ligand HQ^PhtBu^. The reaction was carried out under reflux for 48 h. After cooling to room temperature, a microcrystalline white precipitate was gradually formed and was collected and washed with hot n-hexane. Yield: 82% (0.28 g). M. p. 303°C. Anal. calc. for C_44_H_50_N_4_O_6_Zn: C, 66.37% H, 6.33% N, 7.04% O, 12.06%; found C, 66.31% H, 6.37% N, 7.01% O, 12.11%. IR (*ν*_max_/cm^−1^) 3200–3600br (MeOH^–^N), 2964–2868 (CH_Ali_), 1609s (C=O), 478 (Zn-O). (Supplementary Figure S1A). ^1^H-NMR (500 MHz, DMSO, ppm): 7.97 (4H, d, H^8,8’^), 7.48 (4H, m, H^13,13’^), 7.33 (8H, t, H^14,14’^, H^9,9’^), 7.12 (2H, t, H^10^), 3.35 (6H, s, H^18^ -CH_3_OH),1.64 (6H, s, H^6^ -CH_3_), 1.32 (18H, s, H^17,17’,17″^-C(CH_3_)_3_).

(Supplementary Figure S1B) ^13^C-NMR (500 MHz, DMSO, ppm): 16.96 (C6), 31.53 (C17), 35.05 (C16), 104.22 (C4), 119.50 (C8), 124.37 (C10), 125.19 (C14), 127.61 (C13), 128.89 (C9), 138.83 (C7), 139.71 (C12), 148.34 (C3), 153.21 (C15),166.46 (C5), 190.58 (C11).

### Preparation of the CS@Zn_n_ Films

CS (0.182 g) was dissolved in 13 ml of an aqueous solution containing acetic acid at a ratio of 1% v/v and 10 ml of methanol. The solution was then stirred for 6 h at room temperature until complete dissolution. Films were prepared by adding an appropriate amount of the [Zn(Q^PhtBu^)_2_(MeOH)_2_] complex solubilized in 10 ml of methanol. The resulting solution was added to the CS solution, prepared as previously described, and the mixture was stirred for a further 20 min. The casted CS@Zn_n_ films (*n* = 1.25, 2.5, 5, 7.5, 10% w/w of [Zn(Q^PhtBu^)_2_(MeOH)_2_], in relation to the mass of CS used in the preparation of the film) were prepared by the evaporation-induced method. The above solutions were poured into a Petri dish (9 cm diameter) and then desiccated in an oven at 32°C for 48 h. After drying, the films were peeled off from the casting surface. Thus, six different formulations of CS films with [Zn(Q^PhtBu^)_2_(MeOH)_2_] complex were prepared: five using the specific quantity of [Zn(Q^PhtBu^)_2_(MeOH)_2_] complex and a control formulation consisting of only CS.

### X-Ray Diffraction (PXRD and SCXRD) Analysis

The PXRD patterns of the CS powder, the CS films, and the [Zn(Q^PhtBu^)_2_(MeOH)_2_] complex were acquired on a Bruker D2-Phaser equipped with a Cu Kα radiation (*λ* = 1.5418 Å) and a Lynxeye detector, at 30 kV and 10 mA, with a step size of 0.01° and a step time of 2 s, over an angular range of 5–40° 2θ. In order to determine the crystallinity index (CI) of the CS samples, the powder patterns, after the subtraction of the background, were deconvoluted by applying Gauss fitting procedure using Origin software. The deconvolution process allowed us to identify and separate the crystalline peaks of CS samples. Iterations were repeated until an *R* ^2^ value of 0.998 was reached. CI was calculated according to the following equation:

$$CI=\;\frac{A_{cr}}{A_{tot}}\;\times 100$$

where A_cr_ represents the area under the crystalline peaks and A_tot_ is the total area under the PXRD pattern.

Single-crystal X-ray diffraction data of [Zn(Q^PhtBu^)_2_(MeOH)_2_] complex were collected at room temperature with a Bruker-Nonius X8APEXII CCD area detector system equipped with a graphite monochromator with radiation Mo Kα (*λ* = 0.71073 Å). Data were processed through the SAINT (Bruker 2003) reduction and SADABS (Sheldrick 2003) absorption software. The structure was solved by direct methods and refined by full-matrix least-squares based on F^2^ through the SHELX and SHELXTL structure determination package (Sheldrick 2008). All non-hydrogen atoms were refined anisotropically, and hydrogen atoms were included as the idealized riding atoms. All graphical representations have been obtained by using Olex2 (Dolomanov et al., 2009) and CCDC Mercury 4.3.0. Details of data and structure refinements are reported in Supplementary Table S1. CCDC 2154719 contains the supplementary crystallographic data for this article. These data can be obtained free of charge via http://www.ccdc.cam.ac.uk/conts/retrieving.html.

### Hydrogen Bond Propensity Calculations

HBP calculations have been performed by using the Material Science module available as part of Mercury 2020.1 software from the Cambridge Crystallographic Data Centre (CCDC) with version 5.41 of the Cambridge Structural Database (CSD). The input file used to perform the research was built from the crystallographic data of the molecular fragment of the Zn(II) complex, [Zn(Q^PhtBu^)_2_(MeOH)_2_], to which was added the protonated glucosamine fragment extracted from the CSD (the main repeating unit of ionic CS films). To the resulting target paired molecules of this input file, functional groups were selected as suggested by Mercury, a training dataset (between 500 and 2000 structures per functional group; total hits selected for training dataset 2601, good size) was selected, and the propensity values were calculated using a logistic regression model with an area under ROC curve of 0.83 (“good discrimination”). The propensity score for all donor/acceptor combinations is calculated from the average of the contributing propensity scores, and the coordination score is the average of the coordination scores contributing to the permutation of donors and acceptors. A statistical model based on the likelihood that a functional group participates 0, >1, >2 times is used in the calculation of the coordination scores.

### Opacity Measurement

The film opacity was determined according to the method of Park et al. (Park et al., 2004) by measuring the film absorbance at 600 nm. The CS@Zn_n_ films were cut into 2 cm × 2 cm and directly placed in the spectrophotometer sample chamber. The empty chamber was used as a reference. The opacity of the films was calculated according to the following equation:

$$Opacity=\;\frac{Abs_{600}}{L}$$

where Abs_600_ is the absorbance value at 600 nm and *L* is the film thickness (mm). All measurements were repeated three times.

### Evaluation of the [Zn(Q^PhtBu^)_2_(MeOH)_2_] Release

The release of the complex [Zn(Q^PhtBu^)_2_(MeOH)_2_] from the CS film CS@Zn_10%_ was determined by UV–vis spectroscopy. Circular samples of CS@Zn_10%_ with a diameter of 0.6 cm were immersed in 6.4 ml of PBS (pH 7.4) and kept without stirring for 23 h. Then, absorption spectra of the solution at different immersion times (0, 30, and 180 min) were recorded. For comparison, the same measurements were carried out after immersion of the pure CS film in PBS. The experiments were performed in triplicate. The release of the complex was quantitatively determined by a calibration curve prepared by dissolving [Zn(Q^PhtBu^)_2_(MeOH)_2_] in methanol and then diluted in phosphate-buffered saline (PBS) (methanol 4% v/v) from 8.5·10^−7^ to 1.02·10^−5^ mol L^−1^.

### Antioxidant Activity: 2,2-Diphenyl-1-picrylhydrazyl Radical Scavenging Assay

2,2-Diphenyl-1-picrylhydrazyl radical (DPPH) was dissolved in spectrophotometric grade ethanol to give a 6·10^−5^ M solution. Then, 10 ml of the obtained solution was poured into Petri dishes containing the free [Zn(Q^PhtBu^)_2_(MeOH)_2_] complex (2.3 mg) or the CS films—each incorporating a different amount of [Zn(Q^PhtBu^)_2_(MeOH)_2_]—and left at room temperature in the dark. Absorption spectra of the DPPH solutions were recorded after 3 and 24 h using a Perkin Elmer Lambda 900 spectrophotometer. The antioxidant activity of each sample was calculated by determining the decrease in the optical density at 517 nm according to the following equation:

$$\%\;Antioxidant\;activity=\;\frac{A_{0}-A_{s}}{A_{0}}\cdot 100$$

where A_0_ is the absorbance of the control (DPPH ethanolic solution) and A_S_ is the absorbance of the working solution (DPPH ethanolic solution after incubation with the sample). The test was carried out in triplicate.

To compare the antioxidant activity of the CS@Zn_n_ films with a reference antioxidant sample, CS films loaded with ascorbic acid—CS@AA_n_ (*n* = 1.25 and 10%)—were prepared. In particular, CS@AA_n_ films were prepared as previously described above and loaded with a molar amount of ascorbic acid equivalent to that of [Zn(Q^PhtBu^)_2_(MeOH)_2_] complex in CS@Zn_1.25%_ and CS@Zn_10%_, respectively.

### Antimicrobial Activity

Two bacterial strains, namely, *S. aureus* DSM 346 (Gram-positive) and *E. coli* DSM 1576 (Gram-negative) from DSMZ (German Collection of Microorganisms and Cell Cultures, Braunschweig), were used as model bacteria for the antibacterial activity test of CS films combined with [Zn(Q^PhtBu^)_2_(MeOH)_2_] complex. *S. aureus* was cultured in nutrient medium tryptic soy broth (TSB) and *E. coli* in Luria-Bertani (LB) broth. The organisms were stored at 4°C and subcultured at regular intervals of 30 days to maintain the cell viability. Initially, each strain was cultured in its medium at 37°C for 18 h under 200 rpm rotation. Then, the bacterial cells were collected by centrifugation (4500 rpm, 15 min) and resuspended and diluted in sterile saline solution (0.9% NaCl) to reach approximately 10^8^ cells/ml. The concentrations of bacteria were determined by measuring the optical density at 600 nm. Typically for *E. coli,* a OD600 nm of 0.1 corresponds to a concentration of 10^8^ cells/ml.

### Methods

All materials used in the experiments were autoclaved at 121°C for 25 min to ensure sterility. The antibacterial activity of CS@Zn_n_ films was tested for two bacteria by the agar disk-diffusion method (CLSI 2015). The nutrient agar plate used for experimental (beef extract, peptone, sodium chloride and agar), was prepared spreading uniformly 150 µl of bacterial suspension with a concentration of 10^8^ cells/ml of tested bacterium, with a L-shaped sterile plastic. All types of membranes were cut into circular disks of 6 mm diameter using a circular knife and then, when the inoculum was absorbed, were placed on the agar surface. Each disk was pressed down to ensure complete contact with the agar surface, and then the Petri dishes were inverted and incubated at 35 ± 2°C for 24. Afterward, the plates were examined for the width of inhibition.

## Results and Discussion

### Preparation and Characterization of the [Zn(Q^PhtBu^)_2_(MeOH)_2_] Complex

**FIGURE 1:**
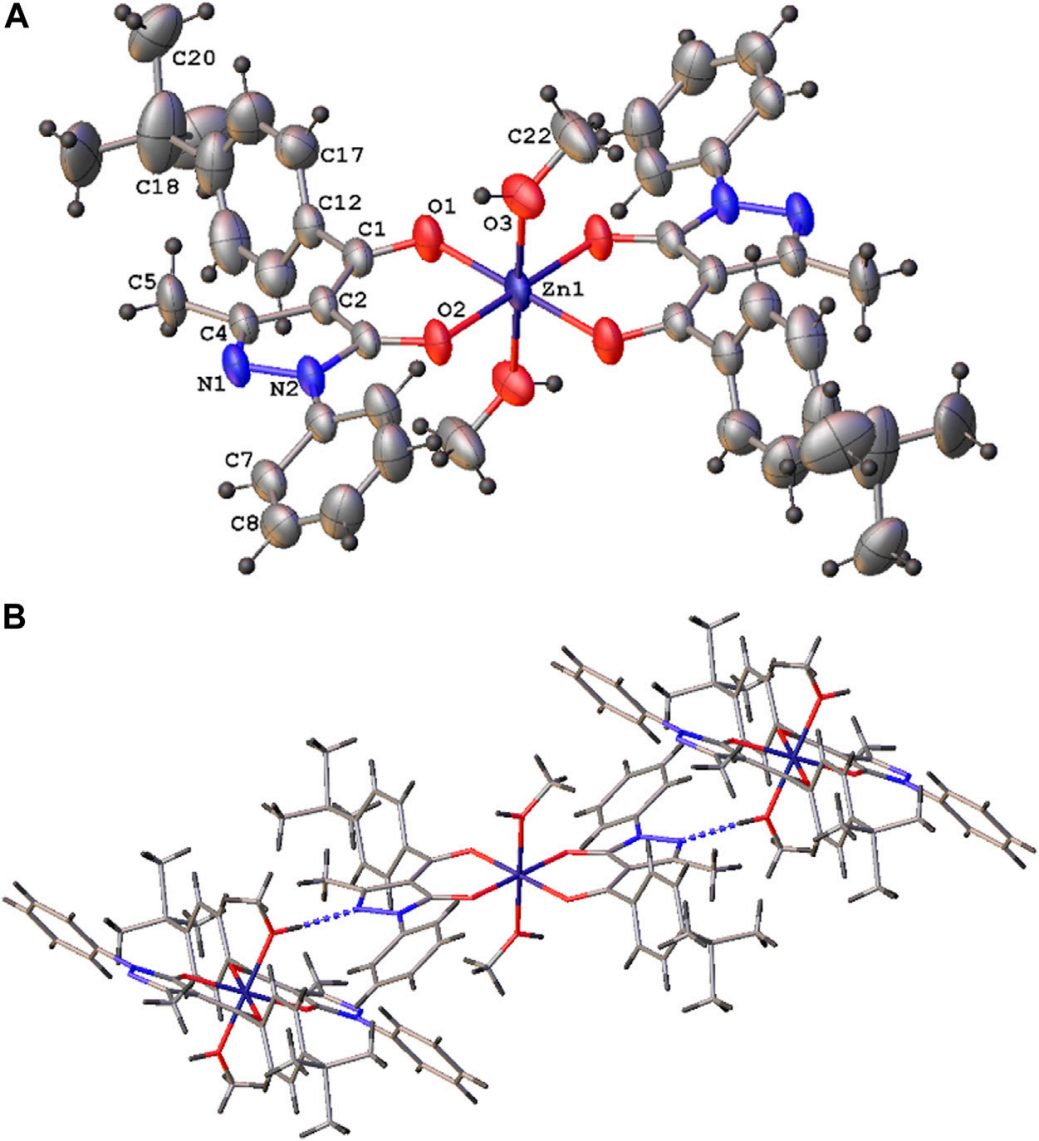
Molecular structure of the [Zn(Q^PhtBu^)_2_(MeOH)_2_] complex **(A)** with atomic numbering scheme and **(B)** crystal packing view showing the predominant O–H–N interactions [O (3)–N (1)^*i*^ 2.798 (2) Å, O (3)-H (3a)...N (1) 164°, *i* = *x*, -*y*+1/2, *z*+1/2].

The [Zn(Q^PhtBu^)_2_(MeOH)_2_] complex has been prepared according to the literature method (Liguori et al., 2010). Both the FT-IR and ^1^H-NMR spectra confirmed the coordination of the acylpyrazolone ligand to the Zn(II) center in the O,O′-bidentate chelating mode (see Experimental Section) as well as the presence of the coordinated methanol molecules also in solution. Moreover, suitable single crystals for X-ray diffraction analysis have been obtained from slow evaporation of the [Zn(Q^PhtBu^)_2_(MeOH)_2_] complex from a methanol solution. The molecular structure of [Zn(Q^PhtBu^)_2_(MeOH)_2_] complex is reported in Figure 1A. Relevant bond distances and angles are reported in Supplementary Table S2. The Zn(II), located on the symmetry inversion center, results in six-coordinated by the O,O bis-chelated HQ^PhtBu^ ligand and the two oxygen atoms of the coordinated methanol molecules. The Zn–O distances are comparable with those found in analogous complexes, while the distance between the Zn(II) and the methanol molecules are relatively elongated with respect to them. The coordination of the HQ^PhtBu^ ligand gives rise to the *anti* isomer, considering *trans* to each other, the oxygen atoms of the acyl moiety, and therefore the relative two substituents.

A relevant structural feature arises from the analysis of the intermolecular interactions existing in the 3D crystal packing. As shown in Figure 1B, each molecule behaves as a hydrogen bond donor and acceptor in the formation of O–H---N hydrogen bonds between the coordinated methanol molecules and the N(1) nitrogen atom of the pyrazole ring of the HQ^PhtBu^-coordinated ligand.

### Preparation and Characterization of CS@Zn_n_ Films

The CS@Zn_n_ films have been obtained as homogeneous, flexible, and transparent films through the solvent casting method, in dilute acidic solution (acetic acid, 1% v/v), using different weight ratios of the [Zn(Q^PhtBu^)_2_(MeOH)_2_] complex to CS. The stability of the [Zn(Q^PhtBu^)_2_(MeOH)_2_] complex in the mixture acetic acid/methanol was monitored spectrophotometrically. As clearly shown in Supplementary Figure S2, the absorption spectra acquired over time (0–72 h) are superimposable, providing evidence of the compound stability. In the solvent casting solution, either the CS or its derivatives CS@Zn_n_ became water-soluble polymers due to the presence of amine groups positively charged after protonation, resulting in relatively hydrophilic charged films. The CS@Zn_n_ films have been characterized by FT-IR, Raman spectroscopy, PXRD, DSC, TGA, and SEM to derive the phase structure and the extent of the interaction between CS and the [Zn(Q^PhtBu^)_2_(MeOH)_2_] additive. Due to the presence of the hydrogen bond acceptor N(1) atom of the pyrazole ring, the -OH donor and acceptor groups of the coordinated methanol molecules, and the donor and acceptor sites of the polymer CS matrix, a complex three-dimensional structure could arise by the effect of a network of intermolecular interactions between CS and the [Zn(Q^PhtBu^)_2_(MeOH)_2_] complex additive.

### Infrared and Raman Spectroscopy

The FT-IR spectrum of the pure CS film is comparable to those reported previously in the literature (Bourtoom and Chinnan 2008; Queiroz et al., 2015). The FT-IR spectrum showed a broad peak between 3500 and 3200 cm^−1^ attributed to the stretching vibration of the free hydroxyl groups, which overlaps the–NH_2_ stretching in the same region. Moreover, the presence of residual N-acetyl groups, since CS is not completely deacetylated, in the CS film was confirmed by the presence of bands at 1647 cm^−1^, 1580 cm^−1^, and 1335 cm^−1^ corresponding, respectively, to the C=O stretching, the N–H bending, and the C–N stretching of the amide group. (Huang et al., 2022). FT-IR spectra of all the CS@Zn_n_ films were recorded and are reported in Supplementary Figure S3. Unfortunately from the FT-IR spectra, regardless of the [Zn(Q^PhtBu^)_2_(MeOH)_2_] complex addition, no significant differences in band shifts have been registered. As the addition of the [Zn(Q^PhtBu^)_2_(MeOH)_2_] complex increases, the appearance of small bands relative to the fingerprint signals of the complex arises from the FT-IR features of the CS matrix, but without any significant shifts suitable to derive any conclusion regarding the eventual interactions between the Zn complex and the polymeric matrix.

**FIGURE 2:**
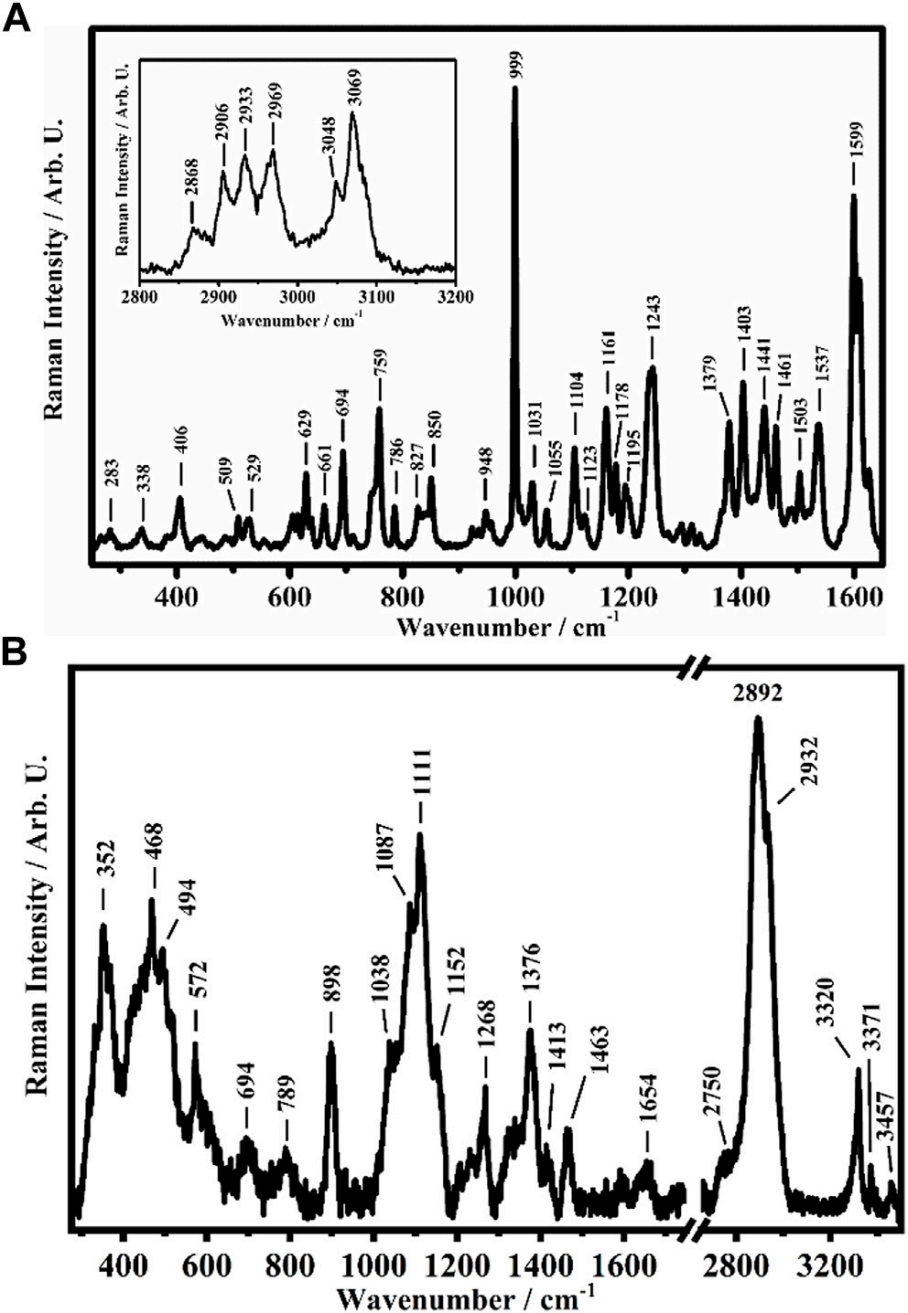
Representative Raman spectra collected on **(A)** the [Zn(Q^PhtBu^)_2_(MeOH)_2_] complex in the solid crystalline phase in the ranges between 250 cm^−1^ and 1,650 cm^−1^ and between 2800 cm^−1^ and 3200 cm^−1^ (in the inset) and **(B)** on CS in the ranges between 275 cm^−1^ and 1750 cm^−1^ and between 2650 cm^−1^ and 3500 cm^−1^.

To investigate the possible interactions between the CS polymer matrix and the Zn complex, all films have been studied though Raman spectroscopy, a powerful tool for understanding of the effects of non-covalent interactions such as hydrogen bonding (Das and Agrawal 2011). The representative Raman spectra collected on a crystalline solid sample of the [Zn(Q^PhtBu^)_2_(MeOH)_2_] complex and on the film of pure CS are shown in Figures 2A,B.

Among the different Raman features present in the spectra shown in Figure 2A of the [Zn(Q^PhtBu^)_2_(MeOH)_2_] complex, the band at 999 cm^−1^ is ascribed to the aromatic ring breathing mode of the acylpyrazolone ligand, while the other bands at 1599 cm^−1^, 1609 cm^−1^, and 1626 cm^−1^ are assigned to the C=O stretching of acylpyrazolone in the enolic forms (Akama et al., 1996; Ueda and Akama 1994). The Raman bands at 2868 cm^−1^, 2906 cm^−1^, 2933 cm^−1^, 2969 cm^−1^, 3048 cm^−1^, and 3069 cm^−1^, shown in the inset of Figure 2A, are assigned to the C–H stretching of the pyrazolone (together with its substituents) ligand of the [Zn(Q^PhtBu^)_2_(MeOH)_2_] complex (Chithambarathanu et al., 2003; Bahgat and EL-Emary 2013).

As shown in Figure 2B, the Raman spectra collected on the pure sample of CS in its film form show features comparable with those reported in the literature (Zhang et al., 2012; Escamilla-García et al., 2013; Kumar and Koh 2013; Mikhailova et al., 2014; Zajac et al., 2015; Menezes et al., 2020). The band at 352 cm^−1^ is attributed to the out-of-plane bending mode (γ) of (OH) and of the pyranoid ring (ϕ), while the two bands at 468 cm^−1^ and 494 cm^−1^ are assigned to the in-plane bending mode (δ) of the glycosidic (C–O–C) and to the δ mode of (CO–NH) + (C–CH_3_), respectively, while the one at 572 cm^−1^ is due to the γ modes of (N–H) and (C=O) and to the out-of-plane bending mode (ω) of (CH_3_). The Raman band centered at 1111 cm^−1^ is attributed to the overlapping of several peaks: two peaks that fall at 1038 cm^−1^ and 1087 cm^−1^, assigned to the deformation mode (ρ) of (CH_3_) and the (δ) modes of (CH) and (OH). Moreover, the stretching (ν) of (C–O–C) + ν(ϕ) + ν(C–OH) + ν(C–CH_2_) + δ(CH) + ρ(CH_2_) + ρ(CH_3_) is found at 1152 cm^−1^, conversely attributed, together with the band at 1087 cm^−1^, by some authors to the stretching modes of the glycosidic bond (C–O–C) and in particular symmetric and antisymmetric of C–O–C (Zhang et al., 2012; Escamilla-García et al., 2013; Kumar and Koh 2013; Mikhailova et al., 2014). The other bands have been assigned as follows: the band at 1268 cm^−1^ is ascribed to the δ(OH–O) hydrogen bond (HB) + ν(C–C) + ν(C–O) + δ(CH) + δ(CH_2_), the band at 1376 cm^−1^ is due to δ(CH_2_), other bands deform to the polysaccharide backbone and to the δ(OH) and ν(ϕ), and the band at 1463 cm^−1^ is assigned to the δ(CH) + ω(CH_2_) modes. The band at 1654 cm^−1^ is due to the double bond C=C. The other bands at 2750 cm^−1^, 2892 cm^−1^, and 2932 cm^−1^ are assigned to the ν(CH), ν(CH_2_), and ν(CH_3_) modes, respectively.

As the main spectral feature, the Raman band at 3320 cm^−1^ is assigned to the N–H stretching, while the other two bands at 3371 cm^−1^ and 3457 cm^−1^ are assigned to the O–H stretching mode (Menezes et al., 2020).

The Raman spectra of the CS@Zn_n_ films show the main characteristics of both [Zn(Q^PhtBu^)_2_(MeOH)_2_] complex and CS matrix (Supplementary Figure S4). In fact, as can be seen in Supplementary Figure S4, the band that falls at 1000 cm^−1^, typical of the [Zn(Q^PhtBu^)_2_(MeOH)_2_] complex, increases, as well as the complex content within the CS matrix increases. However, following the Raman bands ascribable to the CS polymer, the bands above 3300 cm^−1^ seem to decrease as the complex concentration increases. To quantitatively analyze this correlation, the abovementioned bands have been fitted by using Lorentzian functions (Supplementary Figure S5) and the extrapolated intensities used to calculate the variation of their ratios with respect to the Zn complex content.

Supplementary Figures S6A, B show the R_Zn Complex/Chitosan_ and R_(OH)_ ratios (defined as herein reported) plotted as a function of the film composition in percentage:


$$R_{\mathrm{zn}\;complex/chintosam}=\;\frac{I_{(1000cm^{-1})}}{I_{(3300cm^{-1})}+I_{(3320cm^{-1})}}R_{\left(\mathrm{OH}\right)}=\;\frac{I_{(3371cm^{-1})}+I_{(3357cm^{-1})}}{I_{(1000cm^{-1})}}$$

As can be seen in Supplementary Figure S6A, the trend of the R_Zn Complex/Chitosan_ ratio is increasing vs. [Zn(Q^PhtBu^)_2_(MeOH)_2_] complex composition in percentage. However, a jump can be observed for concentrations between 2.5 and 5.0% of the [Zn(Q^PhtBu^)_2_(MeOH)_2_] complex. For this reason, the first two points have been fitted with one linear fit (Supplementary Figure S7), whereas the last three points were fitted by another linear fit (Supplementary Figure S7). The jump and the different slopes found in the linear fits may be due to a change of the structural order within the films induced by the increase of the [Zn(Q^PhtBu^)_2_(MeOH)_2_] complex percentage. The intensities of the bands at 3300 cm^−1^ and 3320 cm^−1^, assigned, as state above, to the N–H stretching, decrease as the Zn complex content in the CS matrix increases. A similar trend has been observed following the intensities of the bands assigned to the hydroxyl group. Indeed, the intensity of the band around 352 cm^−1^, attributed to the out-of-plane bending mode (γ) of (OH) and of the pyranoid ring (ϕ), decreases with the increase of the Zn complex within the film. Moreover, a clear dependence of the R_(OH)_ ratio, calculated by using the sum of the intensities of the bands at 3371 cm^−1^ and 3457 cm^−1^ (assigned to the O–H stretching mode) with the film composition, is evident from Figure 4B. In this case, a significant decrease in the R_(OH)_ value is observed on moving from 1.25 to 2.5% of the [Zn(Q^PhtBu^)_2_(MeOH)_2_] complex content.

All the observed trends seem to indicate that the [Zn(Q^PhtBu^)_2_(MeOH)_2_] complex may simultaneously affect the hydrogen-bonding sites present on the CS structure and interacting itself via hydrogen bond, with the donor and acceptor groups being in the CS backbone structure.

### Powder X-Ray Diffraction Analysis

**FIGURE 3:**
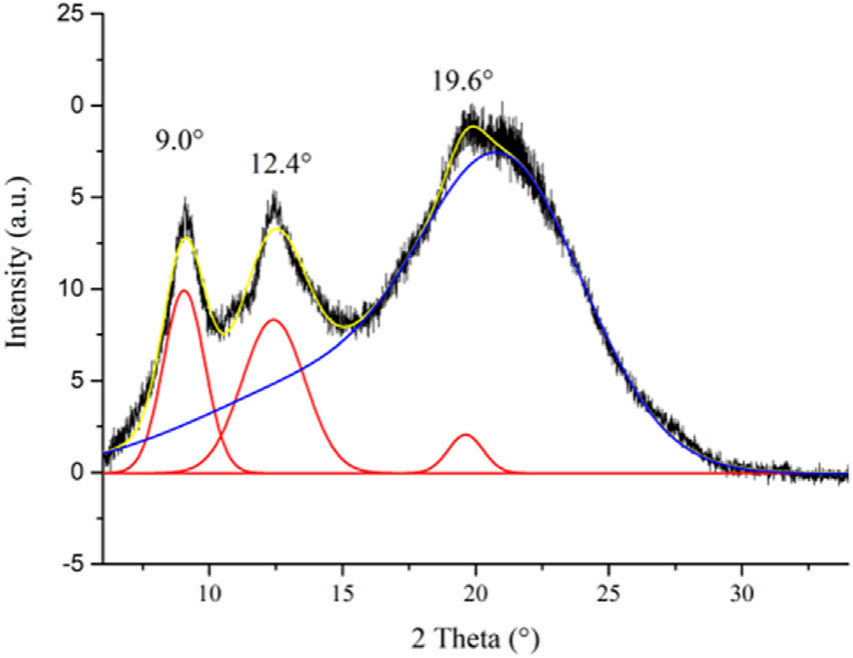
XRD profile of the control CS film with its deconvolution curves (crystalline peaks: red lines; amorphous region: blue curve; cumulative fit curve: yellow line).

CS is a semi-crystalline polymer, presenting both crystalline and amorphous regions. When the CS powder is solubilized in dilute acid solution to prepare ionic CS films, structural changes of the polymeric matrix occur with the variation of the crystallinity degree of CS (Qiao et al., 2021). The CI of a semi-crystalline polymer is related to its stiffness (Corrente et al., 2021); specifically, highly crystalline polymers are characterized by a great rigidity. Therefore, the CI measurement of the CS@Zn_n_ ionic films can provide useful information on their handiness and workability. On this basis, the PXRD analysis of the control CS film (powder and film) was carried out and the CI% values were calculated. The PXRD pattern of the control CS film is characterized by three reflections at 2θ = 9.0°, 12.4°, and 19.6° and a halo centered at 2θ = 22° ca. (Figure 3). These reflections are attributable to the hydrated polymorph of the ionic CS film, with an overall profile appearing as a partially overlapped broad band (Chang et al., 2019; Di Filippo et al., 2020). To separate the crystalline peaks from the amorphous halo, the deconvolution of the PXRD pattern of the control CS film was carried out. The result of the fitting procedure, performed through the Gauss function, is reported in Figure 3. This method allowed to calculate the CI of the sample from the ratio between the area of the three crystalline peaks and the total area of the pattern.

The calculated CI value of 21.2%, as expected, is significantly lower than that of the CS powder sample (CI = 60.8%), measured by applying the same fitting procedure (Supplementary Figure S8). The drastic decrease of crystallinity passing from the CS powder sample to the ionic CS film can be ascribed to the partial disruption of the interchain hydrogen bond network because of the partial protonation of the amino groups and the insertion of acetate anions between the polymeric chains (Misenan et al., 2018).

**FIGURE 4:**
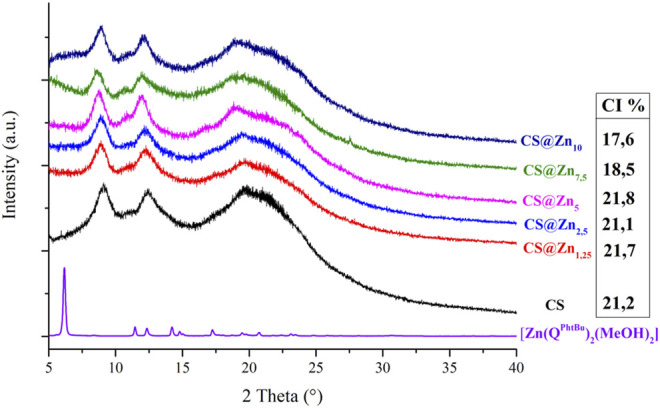
PXRD patterns of the complex [Zn(Q^PhtBu^)_2_(MeOH)_2_] and the ionic CS films and CI% values calculated for the CS films.

The PXRD patterns of the CS@Zn_n_ ionic films have been recorded and compared to that obtained from the pristine CS film, known that the addition of coordination compounds to a polymeric matrix can induce significant structural changes (Scarpelli et al., 2020). As shown in Figure 4, the characteristic reflections of the [Zn(Q^PhtBu^)_2_(MeOH)_2_] complex are absent in the diffractograms of the acetate CS@Zn_n_ films, indicating the deep embedding of the [Zn(Q^PhtBu^)_2_(MeOH)_2_] compound into the polymeric matrix with the formation of highly homogeneous films. Moreover, upon the addition of the [Zn(Q^PhtBu^)_2_(MeOH)_2_] complex to CS, the CI% of the polymer films remains mostly unchanged, as reported in Figure 4, with the exception of the films embedding the highest concentration in the [Zn(Q^PhtBu^)_2_(MeOH)_2_] complex (7.5%, 10%). Indeed, for these concentrated samples, a slight decrease in the CI% is observed (Figure 4). The lower values of CI% measured for these films are reasonably due to the [Zn(Q^PhtBu^)_2_(MeOH)_2_] complex insertion between the polymeric chains with a further decrease of interchain interactions, an effect that becomes evident only at a high concentration of the [Zn(Q^PhtBu^)_2_(MeOH)_2_] complex.

### Thermal Analysis

The thermal decomposition temperature of all CS@Zn_n_ films has been determined by thermogravimetry, and TGA curves are reported in Supplementary Figure S9A. Regarding the pure CS film, an initial weight loss from 40 to 100°C is observed, which is due to the removal of moisture and eventual solvent residue present in the polymeric matrix. The second significant weight loss is in the range of temperature between 150 and 270°C, a thermal event related to the evaporation of structurally bound water molecules entrapped within the film network (Shen and Kamdem 2015; Grande Tovar et al., 2020). At 275°C, the thermal decomposition including the dehydration of the saccharide rings, depolymerization, and loss of acetylated and deacetylated units of the polymer is then initiated.

All CS@Zn_n_ films show a similar thermal behavior when compared to the pure CS film. However, it is clearly evident that the loss of structurally bound water at 150–290°C, present in pure CS, is now completely absent in all thermograms, while the first loss at 40–100°C is at present more pronounced when compared to pure CS. This observation seems to point out that incorporating the [Zn(Q^PhtBu^)_2_(MeOH)_2_] complex to CS displaces the structurally bound water molecules, which become less bonded to the polymeric chains, hence behaving as a mere moisturizing agent. Moreover, the [Zn(Q^PhtBu^)_2_(MeOH)_2_] complex contributes to a slight improvement in the overall thermal stability of the CS films, since the first decomposition starting at 275°C in the pure CS film is shifted at 315°C in the case of the Zn-containing samples.

DSC thermograms of CS and CS@Zn_n_ films are shown in Supplementary Figure S9B. They clearly show that all CS and CS@Zn_n_ films exhibit similar features. One broad endothermic peak between 50 and 110°C is related to dehydration and probably to the evaporation of residual solvent that was used during the preparation of the films. The CS@Zn_n_ films then display an exothermic peak from 250 to 300°C that is relative to the oxidation of free amine units together with the initial thermal decomposition (Sreenivasan 1996; Deng et al., 2007). Noteworthy, for all CS@Zn_n_ films, no endothermic peak at 303°C has been recorded that corresponds to the melting point of the [Zn(Q^PhtBu^)_2_(MeOH)_2_] complex. This is in accordance with the complete amorphous state of the [Zn(Q^PhtBu^)_2_(MeOH)_2_] complex that is reached once incorporated within the polymeric matrix.

### Film Morphology and Thickness

**FIGURE 5:**
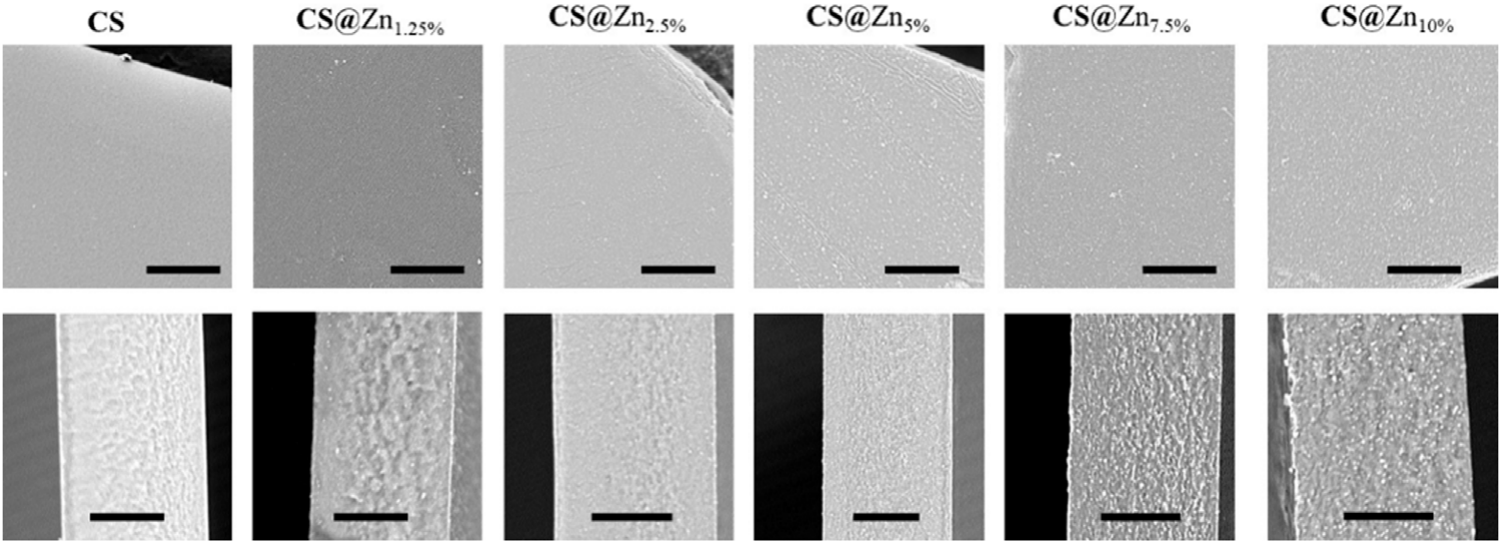
SEM images of the prepared films, up: images of the surface; down: corresponding cross section (scale bars 10 μm).

According to the acquired SEM images, reported in Figure 5, all prepared films display a rather smooth surface, and their cross sections reveal a dense and compact morphology, without pores or holes. However, as the concentration of the [Zn(Q^PhtBu^)_2_(MeOH)_2_] complex increases, homogeneously distributed spherical aggregates of small dimensions (from 0.1 to 0.5 μm in diameter) are observable both in surface and in section, which are easily identified on the images as white spots. Their number increases with the increase of the [Zn(Q^PhtBu^)_2_(MeOH)_2_] complex concentration, but their size roughly remains unvaried. Although film “burning” was observed during the punctual EDX analysis, owing to the organic nature of the sample and the necessity to reach higher voltages, zinc together with carbon, oxygen, and nitrogen was clearly detected, which is indicative of the complex content of these aggregates. According to the PXRD patterns reported in Figure 4, these aggregates must therefore be amorphous in state, and they probably result from a segregation process occurring during the evaporation of the solvent while the films are prepared. Noteworthy, the high content in O and C elements also detected by EDX with respect to Zn together with the fact that the white spots suffer from decomposition when irradiated for punctual EDX analysis would suggest that these aggregates also contain an important part of the polymeric chains (Supplementary Figure S10). It can therefore be hypothesized that interactions are instituted between the complex molecules and the CS functional groups that promote segregation and encapsulation of the complex, thus preventing its crystallization but leading nevertheless to a higher content in the Zn complex in these amorphous aggregates with respect to the bulk of the film. All CS@Zn_n_ films display an average thickness of 20 μm, when sampled in the center of the prepared membrane, and this thickness value is maintained for samples taken as far as 3 cm from the center. Beyond that point, film thickness gradually increases to reach around twice its value on the border of the prepared membrane, consistent with the solvent casting method employed and therefore with an accumulation of material along the edges of the Petri dish used for casting.

### Film Opacity

Opacity of the CS@Zn_n_ films was determined by UV-spectroscopy by measuring the resulting absorbance at 600 nm wavelength. To ensure consistency of the data, all measured absorbance data were corrected by the exact film thickness measured by SEM cross-sectional images of the sample prior to UV analysis, as reported in Supplementary Figure S11.

**FIGURE 6:**
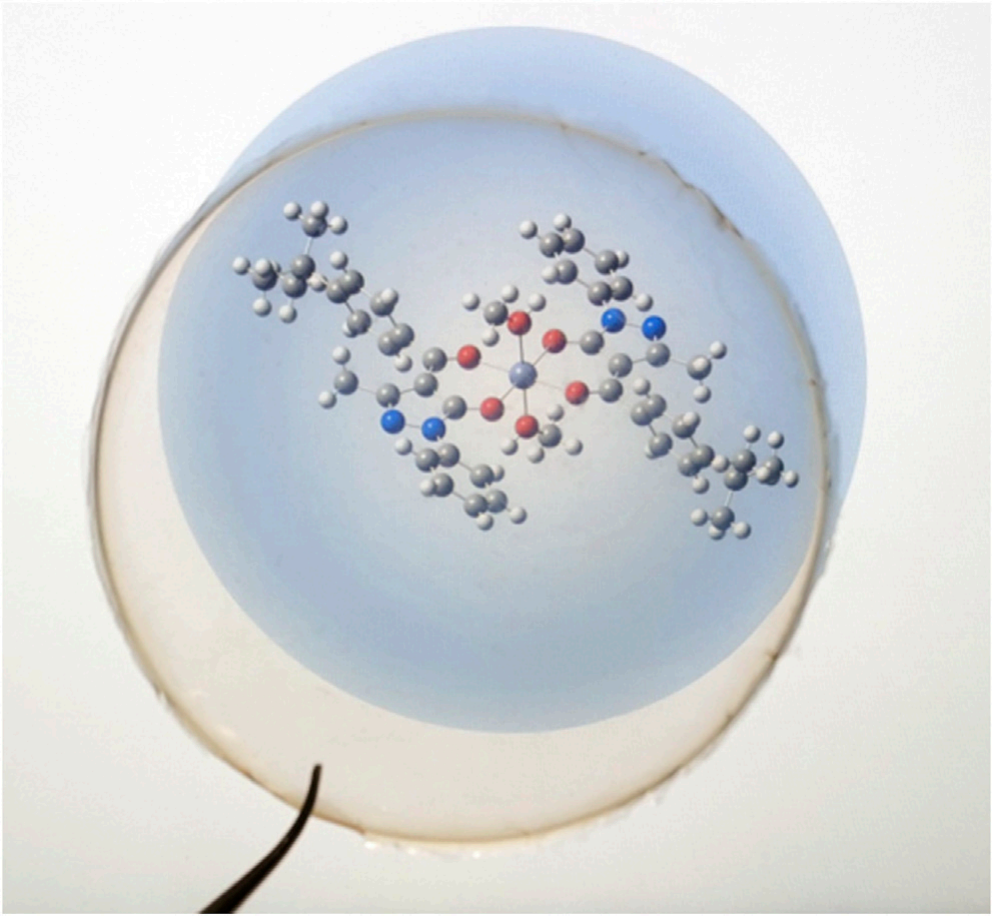
Photograph of the CS@Zn_10%_ film on the molecular structure of the [Zn(Q^PhtBu^)_2_(MeOH)_2_] complex.

In general, high transparency and lightness are desirable for film coating and packaging use. Film opacity is a valuable property that may influence the consumer acceptability of a product (Dong et al., 2022). On the other hand, a high value of opacity is a favorable feature that could make films more efficient both in the light barrier and in preventing photochemical reactions caused by UV irradiation. In the case of CS@Zn_n_ films, compared with the pure CS film, an increase in the [Zn(Q^PhtBu^)_2_(MeOH)_2_] complex content resulted in an increase in the opacity value, probably due to an increase in the density of the network structure obtained through interaction between the Zn complex and the polymeric matrix (Zhang et al., 2020). Nonetheless, the presence of the Zn(II) complex into the CS films resulted in a light pink coloration, and even at a highest concentration (CS@Zn10%), the film is still transparent enough to allow the perfect reading of writings placed under it, as can be observed clearly in the photographs reported in Figure 6.

### HBP Analysis: Evaluation of a Mode of Interactions Between [Zn(Q^PhtBu^)_2_(MeOH)_2_] and CS in CS@Zn_n_ Films

**FIGURE 7:**
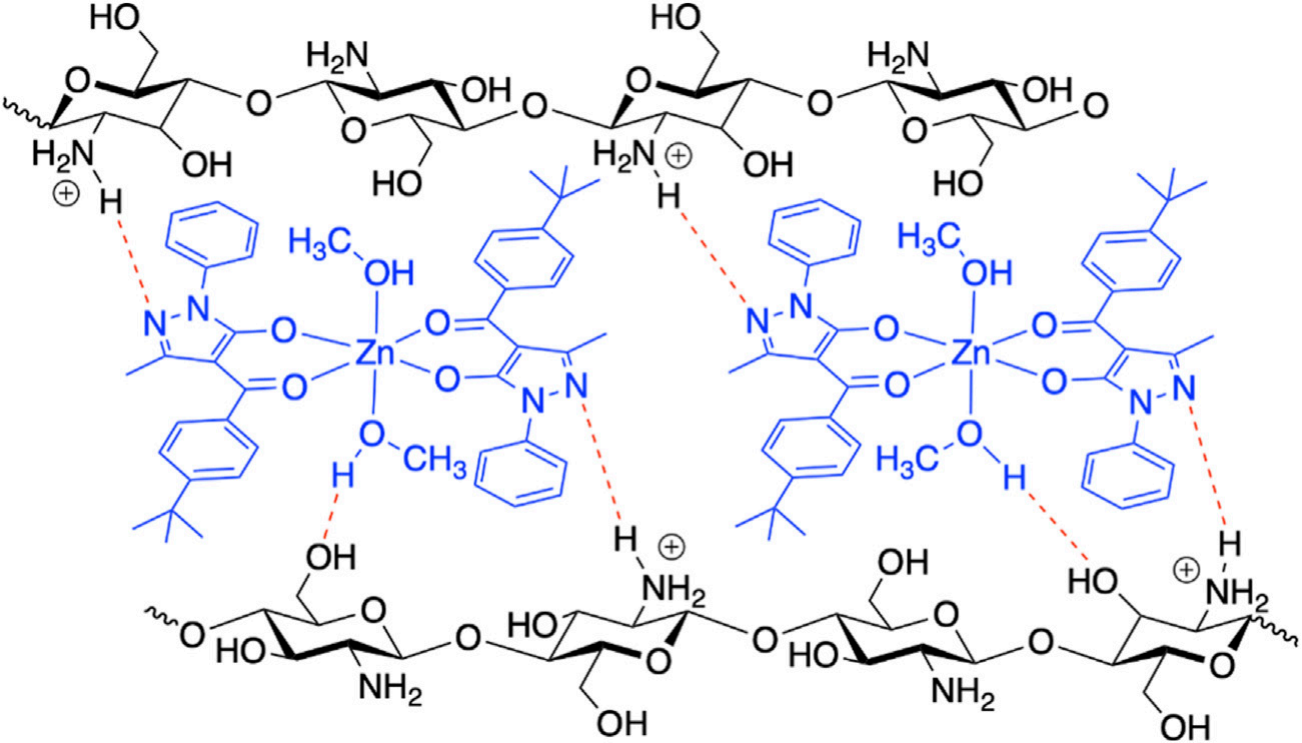
Proposed model scheme of the plausible intermolecular hydrogen bonds between [Zn(Q^PhtBu^)_2_(MeOH)_2_] and CS in the formation of the CS@Zn_n_ films.

In order to investigate the possible modes of interaction between [Zn(Q^PhtBu^)_2_(MeOH)_2_] and CS, an input file comprising the Zn(II) complex and a protonated glucosamine fragment (the main repeating unit of the ionic CS films) was used as target paired-molecules to calculate Hydrogen Bond Propensity, through the routine available in the CCDC software Mercury 2020.1 (details of the input file creation are reported in the Experimental Section) (Galek et al., 2007; Macrae et al., 2008; Galek et al., 2014; Mazzone et al., 2021). Indeed, considering the multiple potential hydrogen-bonding groups present in both molecules, shown in Supplementary Figure S9, it can be assumed that the Zn(II) complex and CS interact through the formation of hydrogen bonds, which can be predicted through the HBP tool. The –NH_3_^+^ group of glucosamine (defined as cyclic_T4C_nh3) has been identified as a donor, whereas the N(1) atom of the pyrazole rings (Figure 1A; indicated as T2NH0_cyclic), the O(1) and O(2) of the Zn(II) complex (T2OH0 fragments), together with the other O atom of glucosamine (al_hydroxy_6), have been selected as acceptors. The numerous hydroxyl groups present within the model (al_hydroxy_3, acyclic_al_oh, and methanol_2 fragments) can act as both donors and acceptors. The main interactions predicted through the HBP tool are reported in Supplementary Table S3. The results of the calculations show that the interaction observed in the crystal structure of [Zn(Q^PhtBu^)_2_(MeOH)_2_] between the hydroxyl group of the methanol molecules coordinated to the metal center and the N(1) atom of the pyrazole rings displays, as expected, a high propensity (0.89). However, according to the HBP calculations, this interaction competes with other hydrogen bond pairings within the target paired-molecules. In particular, methanol molecules exhibit elevated affinity toward the al_hydroxy_3 group and the acyclic_al_oh group of the glucosamine fragment (propensity values of 0.92 and 0.86, respectively). Moreover, the N(1) atom of the pyrazole rings has a high propensity (0.86) to interact with the –NH_3_^+^ group of the glucosamine molecule. Thus, although the propensity values of the pairs methanol_2/T2NH0_cyclic and cyclic_T4C_nh3/2NH0_cyclic are similar, it is reasonable to assume that, within the target paired-molecules, the establishment of multiple interactions between the [Zn(Q^PhtBu^)_2_(MeOH)_2_] complex and the glucosamine fragment is favored over the single hydrogen bond between methanol_2 and T2NH0_cyclic, observed in the crystal structure (Figure 7). Therefore, it can be hypothesized that when the Zn(II) complex is embedded within the ionic CS polymeric matrix, the intermolecular hydrogen bonds between methanol and N(1) atom break and are replaced by the interactions between the N(1) atom and the –NH_3_^+^ groups; simultaneously, the two methanol molecules coordinated to the metal center interact with both the hydroxyl group of the glucosamine and the hydroxymethyl group of another glucosamine fragment, resulting in a perfect embedding of the metal complex into the ionic CS network.

### Evaluation of the [Zn(Q^PhtBu^)_2_(MeOH)_2_] Release

UV–vis spectroscopy was used to monitor and quantify the release of [Zn(Q^PhtBu^)_2_(MeOH)_2_] from the CS@Zn_10%_ sample. A circular piece of CS film (control) or CS@Zn_10%_ film was immersed in PBS (pH 7.4), and the electronic spectra of the solution were recorded at different immersion times (Supplementary Figure S13). As shown in Supplementary Figure S13A, the absorption spectra of the PBS solution after immersion of the CS@Zn_10%_ sample display a broad band (320–400 nm) typical of the [Zn(Q^PhtBu^)_2_(MeOH)_2_] complex. Conversely, no absorption was observed in the same spectral region for the control sample. This result clearly highlights the ability of the CS film to deliver the compound when exposed to aqueous solution. Moreover, no change in absorbance was observed over time, suggesting that—after 180 min of monitoring—the amount of the released complex remains almost constant. In order to determine the concentration of the compound released into the solution, a calibration curve (Supplementary Figure S13B) was carried out by solutions of [Zn(Q^PhtBu^)_2_(MeOH)_2_], obtaining a value of 2.36 ⋅10^−6^ mol L^−1^. Considering the total amount of the [Zn(Q^PhtBu^)_2_(MeOH)_2_] complex present in the evaluated CS@Zn_10%_ film (0.097 mg), only a small fraction of compound is released into the aqueous medium (0.012 mg).

### Antioxidant and Antimicrobic Properties Antioxidant Activity

2,2-Diphenyl-1-picrylhydrazyl (DPPH) scavenging method was used to evaluate the antioxidant activity of the CS and CS@Zn_n_ film samples (Blois 1958). DPPH is a stable radical molecule, and due to the delocalization of the spare electron on the whole molecule able to give rise to a strong absorption in the green region of the visible spectrum, its ethanol solution appears deep purple in color (Kedare and Singh 2011). In the presence of antioxidant molecules—hydrogen or electron donors—the odd electron of the radical becomes paired off resulting in a decrease of the optical density at 517 nm and a color change of the solution from purple to pale yellow with respect to the number of electrons caught.

**FIGURE 8:**
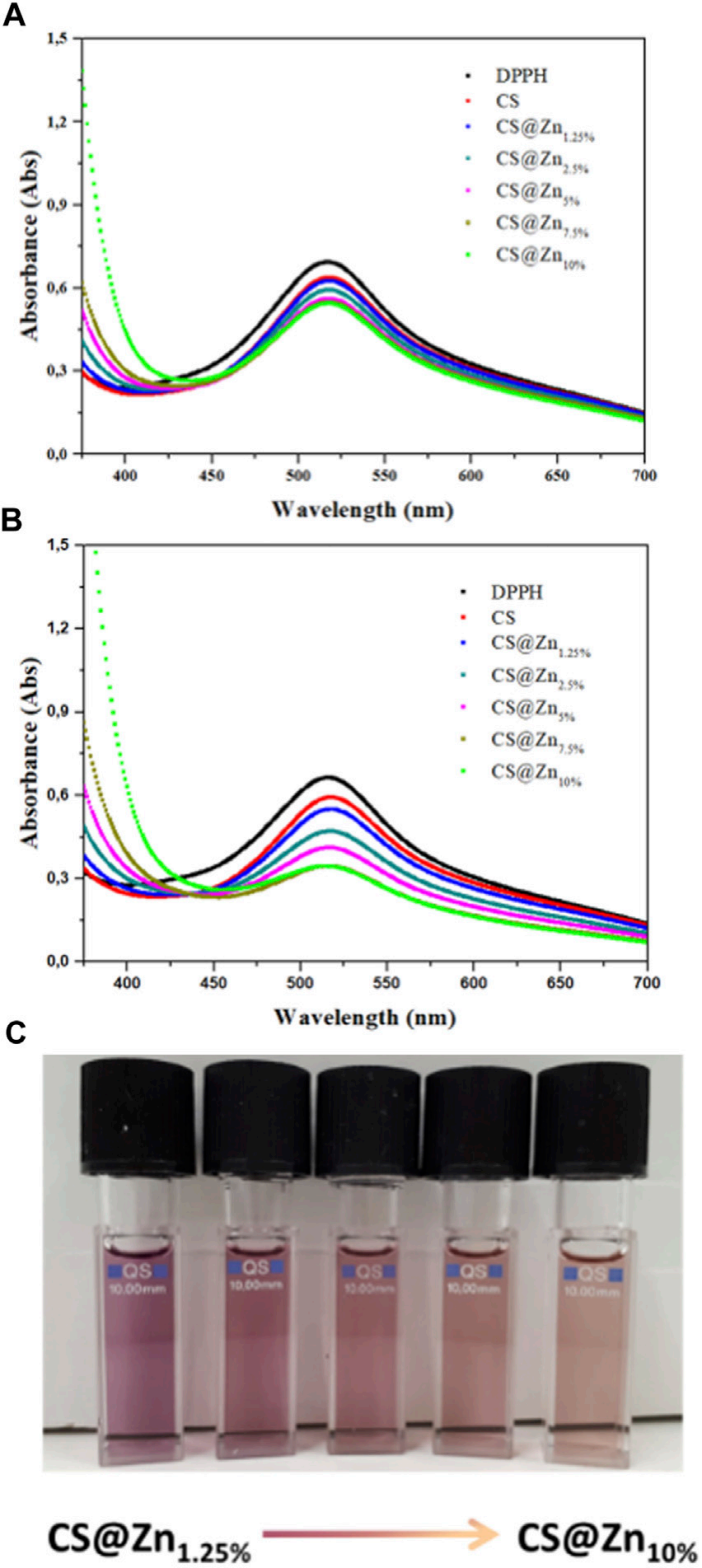
Absorption spectra of DPPH ethanolic solution (control) and after incubation for 3 **(A)** and 24 h **(B)**, with chitosan films incorporating different amounts of the [Zn(Q^PhtBu^)_2_(MeOH)_2_] complex. **(C)** Picture of DPPH working solutions after 24 h of incubation with the samples CS@Zn_n_. The discoloration of the solution is proportional to the antioxidant ability of the sample and increases with the increasing [Zn(Q^PhtBu^)_2_(MeOH)_2_] complex loading rate.

The absorption spectra of DPPH in the ethanol solution and after incubation with the [Zn(Q^PhtBu^)_2_(MeOH)_2_] complex and CS and CS@Zn_n_ films were recorded at room temperature after 3 and 24 h (Figure 8 and Table 1) to assess a potential time-dependence of the DPPH radical scavenging activity. The latter was expressed as the percentage reduction of the absorbance values of the initial DPPH solution (Table 1). As reported in Table 1 and Supplementary Figure S14, the inhibition of the DPPH radical after 3 h of incubation with the free [Zn(Q^PhtBu^)_2_(MeOH)_2_] complex was of 2.82 ± 0.12% and it did not increase significantly, extending the incubation period to 24 h (3.14 ± 0.14%).

**TABLE 1:** DPPH scavenging ability of [Zn(Q^PhtBu^)_2_(MeOH)_2_], CS, CS@AA_n_ (AA = ascorbic acid; *n* = 1.25 and 10%), and CS@Zn_n_ films.

Sample	Antioxidant Activity (%) 3 h	Antioxidant Activity (%) 24 h
[Zn(Q^PhtBu^)_2_(MeOH)_2_]	2.82 ± 0.12	3.14 ± 0.14
CS	7.94 ± 0.22	10.85 ± 0.11
CS@AA_1.25%_	8.09 ± 0.31	12.82 ± 0.22
CS@Zn_1.25%_	9.68 ± 0.37	17.34 ± 0.18
CS@Zn_2.5%_	14.45 ± 0.15	29.11 ± 0.31
CS@Zn_5%_	19.07 ± 0.45	38.15 ± 0.26
CS@Zn_7.5%_	20.8 ± 0.14	47.96 ± 0.52
CS@Zn_10%_	21.53 ± 0.34	48.26 ± 0.47
CS@AA_10%_	11.27 ± 0.15	28.20 ± 0.12

A higher DPPH scavenging activity than that of the free [Zn(Q^PhtBu^)_2_(MeOH)_2_] was observed for the pure CS film (Table 1 and Figure 8), reaching values after 3 and 24 h of 7.94 ± 0.22% and 10.85 ± 0.11%, respectively, in agreement with those reported in previous studies (Moradi et al., 2012; Jiang et al., 2021; Kadam et al., 2021). In the CS@Zn_n_ films, a combination of the antioxidant properties of the [Zn(Q^PhtBu^)_2_(MeOH)_2_] complex with those of the pure CS film is observed. As shown in Figure 8A, after 3 h, the DPPH absorption band centered at 517 nm decreases, highlighting the antioxidant ability of the CS@Zn_n_ films with an enhancement in the scavenging activity as the concentration of the loaded [Zn(Q^PhtBu^)_2_(MeOH)_2_] complex increases (Table 1). After 24 h (Figure 8B), the results clearly indicate that all CS@Zn_n_ films exhibited a higher antioxidant activity against DPPH, CS@Zn_10%_ displaying the highest antioxidant ability with a value of 48.26% (Table 1 and Figure 8C). Then, to compare the antioxidant activity of the CS@Zn_n_ films with a reference antioxidant sample, CS films loaded with ascorbic acid were prepared and the scavenging properties were tested (Supplementary Figure S15). In particular, CS@AA_n_ (*n* = 1.25 and 10%) films were loaded with a molar amount of ascorbic acid equivalent to that of the [Zn(Q^PhtBu^)_2_(MeOH)_2_] complex in CS@Zn_1.25%_ and CS@Zn_10%_, respectively.

As reported in Table 1, while the DPPH scavenging activity of pure CS is almost comparable with that of the CS@AA_n_ films after both 3 and 24 h, the effect of the introduction of the [Zn(Q^PhtBu^)_2_(MeOH)_2_] complex into the polymeric matrix is evident even at the lowest concentration. Indeed, the DPPH scavenging activity observed after 3 and 24 h for CS@Zn_1.25%_ and CS@Zn_10%_ is found higher than that observed for ascorbic acid CS films, highlighting a better antioxidant activity induced by the addition of the [Zn(Q^PhtBu^)_2_(MeOH)_2_] complex.

### Antibacterial Activity

It is well known that one of the most important properties of CS and its derivatives is its antibacterial activity. The antibacterial action of CS is strongly influenced by many factors, the intrinsic ones being the positive charge distribution, the MW, the concentration, the hydrophilic/hydrophobic, and chelating capabilities. In this scenario, considering that the CS used in the preparation of the CS@Zn_n_ films has a high degree of deacetylation (90%) and dilute acidic water solution (acetic acid, 1% v/v) has been used in order to obtain positively charged polymeric films, conditions that should enhance the electrostatic interaction between the polycationic structure and the anionic components on the surface of the microorganisms, the effects of the addition of the [Zn(Q^PhtBu^)_2_(MeOH)_2_] complex are investigated without further modification of the backbone charged structure of the CS matrix. The antimicrobial properties of the CS@Zn_n_ films are assessed by the agar disk-diffusion method measuring the diameter of the zone of complete inhibition (clear zone) around the film samples and below, exactly at the contact area of these films with the agar surface in the case of Gram-positive (*S. aureus*) and Gram-negative (*E. coli*) bacteria. This method has been chosen to ensure the evaluation of the antimicrobial action of the new zinc-containing derivative films in their intact solid form, avoiding swelling and eventual hydrogel formation in water and physiological conditions.

**FIGURE 9:**
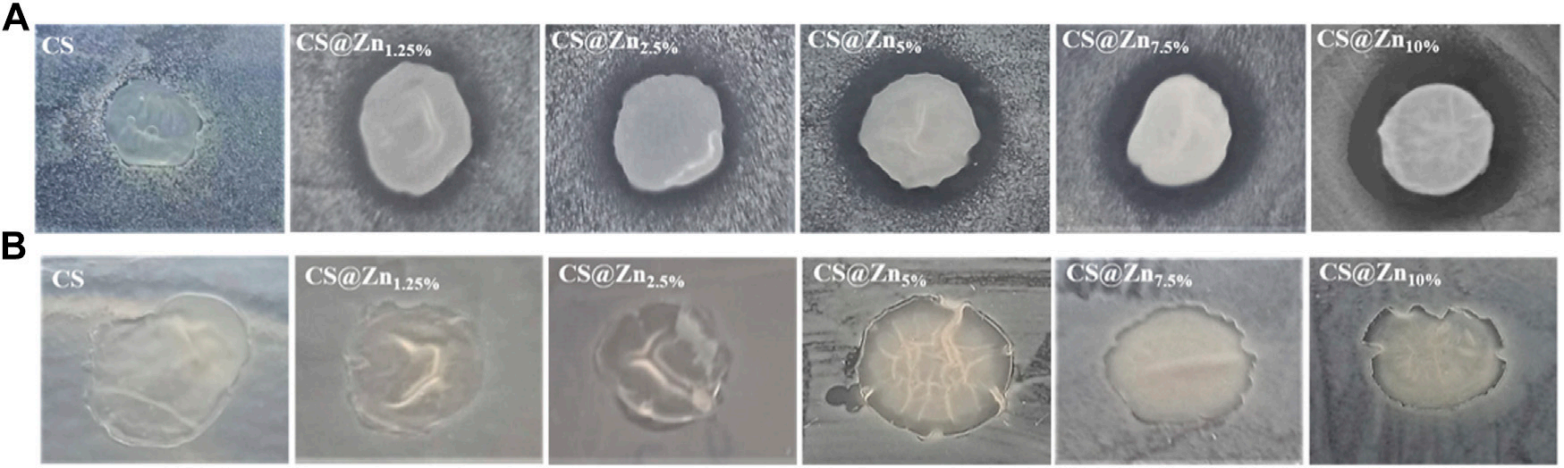
Inhibitory effect of CS and CS@Zn_n_ films on the growth of *Staphylococcus aureus* **(A)** and *Escherichia coli* **(B)**.

The pictures of diffusion assays resulting from CS and CS@Zn_n_ films with different concentrations of the [Zn(Q^PhtBu^)_2_(MeOH)_2_] complex in contact with the two microorganisms are shown in Figure 9. Each inhibition zone has been measured in triplicate with a caliper and the data reported as the average of four separate experimental runs (Table 2).

**TABLE 2:** The diameter (mm) of the inhibition zone of CS and CS@Zn_n_ films including the diameter of the disk (6 mm). The data are reported as the average of four determinations ±SD.

Film Type	Chitosan/Zn Complex % (p/p)	Diameter of Inhibitory Zone *S. aureus* (mm)	Diameter of Inhibitory Zone E. coli (mm)
CS	0%	7.0 ± 0,8	7.0 ± 0.6
CS@Zn_1.25%_	1.25%	9.0 ± 0.2	7.2 ± 0.4
CS@Zn_2.5%_	2.5%	9.9 ± 0.5	7.5 ± 0.2
CS@Zn_5%_	5%	10.9 ± 0,4	7.5 ± 0.1
CS@Zn_7.5%_	7.5%	11.4 ± 0.1	7.8 ± 0.2
CS@Zn_10%_	10%	11.9 ± 0.5	7.9 ± 0.2

As shown in Figure 9, no significant inhibition halos around the CS and CS@Zn_n_ films appear when they are in contact with both the pathogens analyzed, even if no bacterial growth under the whole film surface is observed. Several authors found similar results analyzing the antimicrobial activity of CS films against various microorganisms through the agar disk-diffusion method. It has been reported that no inhibitory halos are observed against any microorganism, CS, in film form, is unable to diffuse through the adjacent agar media, and only organisms in direct contact with the active sites of the film are inhibited (Coma et al., 2002; Pranoto et al., 2005). However, despite this limitation, the CS@Zn_n_ films combined with the [Zn(Q^PhtBu^)_2_(MeOH)_2_] complex show an evident increase in their antimicrobial activity against *S. aureus*, with a clear concentration-dependence (Table 2). No significant changes with respect to the pure CS film are observed when CS@Zn_n_ films are in contact with *E. coli* bacteria, proving that the embedding of the [Zn(Q^PhtBu^)_2_(MeOH)_2_] complex into the CS polymeric matrix makes the derived films more effective against Gram-positive than Gram-negative bacteria. Moreover, according to the inhibition zone diameters, the antibacterial activity against Gram-positive microorganism seems strictly related to the amount of the [Zn(Q^PhtBu^)_2_(MeOH)_2_] complex, increasing at a higher concentration of the additive (Supplementary Figure S16).

The difference in the antimicrobial activity of the films against the analyzed bacteria can be related to the difference in their structural and chemical cell membrane compositions. Gram-positive bacteria have a cell wall primarily made up of a peptidoglycan layer as well as teichoic and lipoteichoic acids. The cell wall of Gram-negative bacteria is more complex due to the presence of an outer membrane, which is composed mainly of lipopolysaccharide (LPS), in addition to a thin peptidoglycan layer. In Gram-positive bacteria, the adsorption of the biocidal molecules occurred on the lipoteichoic acid layer, which is characterized by a charged nature and the ability to interact with the biocide molecules. While in the Gram-negative bacteria, the lipid layer (highly nonpolar layer) is the target of the biocide molecules and this outer membrane on the Gram-negative cell wall confers more resistance than the thicker peptidoglycan layer from Gram-positive bacteria.

## Conclusion

Essential transition metal ions and their metal complexes are nowadays considered useful and effective additives in the improvement of the antibacterial and antioxidant activities of CS and its derivatives in their film formulation. Moreover, CS due to its biodegradability, biocompatibility, and non-toxicity is receiving special attention in the biomedical area as a selective drug delivery system. Generally, the incorporation of metal ions into CS and its derivative polymers modified their structural backbone by complexation interactions, achieved *via* either the CS N- and/or O-functional groups or suitable ligands specifically covalently bound on the CS polymer chain. In this article, a newly synthesized acylpyrazolonate Zn(II) complex, [Zn(Q^PhtBu^)_2_(MeOH)_2_], has been used as a biologically active additive in the formation of a series of CS@Zn_n_ films with a different content of Zn complex. The X-ray single-crystal structural analysis of [Zn(Q^PhtBu^)_2_(MeOH)_2_] has highlighted the octahedral geometry around the Zn(II) metal ion, with the two O,O-chelated HQ^PhtBu^ ligands laying on the basal plane and two coordinated methanol molecules above and below of it. From the analysis of the intermolecular interactions, it has been observed that each molecule can behave as a hydrogen bond donor and acceptor in the formation of O-H---N hydrogen bonds through the coordinated methanol molecules and the nitrogen atom of the pyrazole ring. These structural features have been found particularly relevant in the formation of the CS@Zn_n_ homogeneous, flexible, and transparent films, obtained through the solvent casting method, in dilute acidic solution (acetic acid, 1% v/v), using different weight ratios of the [Zn(Q^PhtBu^)_2_(MeOH)_2_] complex to CS. Indeed, most probably, no CS–Zn complexation occurs, but from almost all the physicochemical characterization of the CS@Zn_n_ films, the segregation and encapsulation of the complex into the CS polymeric matrix is based on the intermolecular interactions instituted between the complex molecules and the CS functional groups. Through the HBP analysis, based on the likelihood of competitive interactions in the target paired-molecules that build up with the Zn(II) complex and a protonated glucosamine fragment (the main repeating unit of ionic CS films), screening of the most probable interacting couples has been conducted. As a result, the intermolecular hydrogen bonds between the coordinated methanol molecules and the nitrogen atom of the pyrazole ring present in the crystal structure of [Zn(Q^PhtBu^)_2_(MeOH)_2_] break, being replaced by N–H---N and O–H---O hydrogen bonds between the Zn(II) complex and the –NH_3_^+^, hydroxyl and hydroxymethyl groups of the glucosamine fragment. The antioxidant activity of the CS@Zn_n_ films at different contents of the [Zn(Q^PhtBu^)_2_(MeOH)_2_] complex has been evaluated according to the DPPH method. In the CS@Zn_n_ films, the combination of the antioxidant activity of the [Zn(Q^PhtBu^)_2_(MeOH)_2_] complex with those of pure CS film results in an enhancement in the scavenging activity even at the lowest concentration of the Zn(II) complex into the polymeric matrix and in its gradual increase as the concentration of the loaded [Zn(Q^PhtBu^)_2_(MeOH)_2_] complex increases. While the pure CS film shows the DPPH scavenging activity comparable with that of the ascorbic acid (AA) CS film at the lowest concentration of AA, both CS@Zn_1.25%_ and CS@Zn_10%_ show significantly better antioxidant activity, clearly proving the decisive role played by the Zn(II) complex. Moreover, the CS@Zn_n_ films were tried out as antimicrobial agents, showing an increase in the antimicrobial activity against Gram-positive bacteria (*S. aureus*) with respect to pure CS, with a clear concentration-dependence, when detected by the agar disk-diffusion method. No significant changes with respect to pure CS film are observed when CS@Zn_n_ films are in contact with *E. coli* bacteria, meaning proving that the embedding of the [Zn(Q^PhtBu^)_2_(MeOH)_2_] complex into the CS polymeric matrix makes the derived films more effective against Gram-positive than Gram-negative bacteria. In conclusion, the bioactivity shown by the new CS@Zn_n_ films makes them attractive for practical applications, for which further studies on their safety profile and, specifically, on the safety of the Zn(II) complex as a bioactive additive are still underway.

## Data Availability

The original contributions presented in this study are publicly available. This data can be found here: https://www.ccdc.cam.ac.uk/structures/2154719.
