# Supplementary material for: New Zinc-Based Active Chitosan Films: Physicochemical Characterization, Antioxidant, and Antimicrobial Properties
Source: Front Chem. 2022 May 31;10:884059. doi: 10.3389/fchem.2022.884059 (PMC9194505; doi:10.3389/fchem.2022.884059)
Supplement: Supplementary file 1 [file DataSheet2.pdf]

Supplementary Material

a)

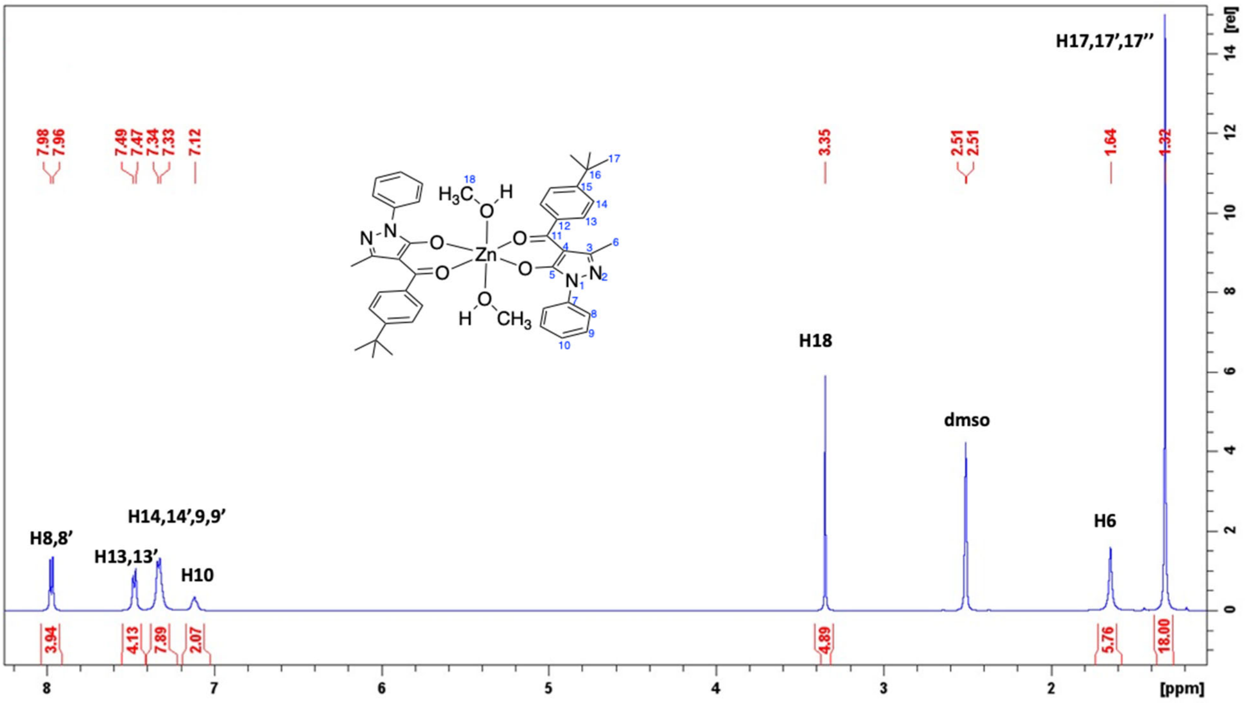

b)

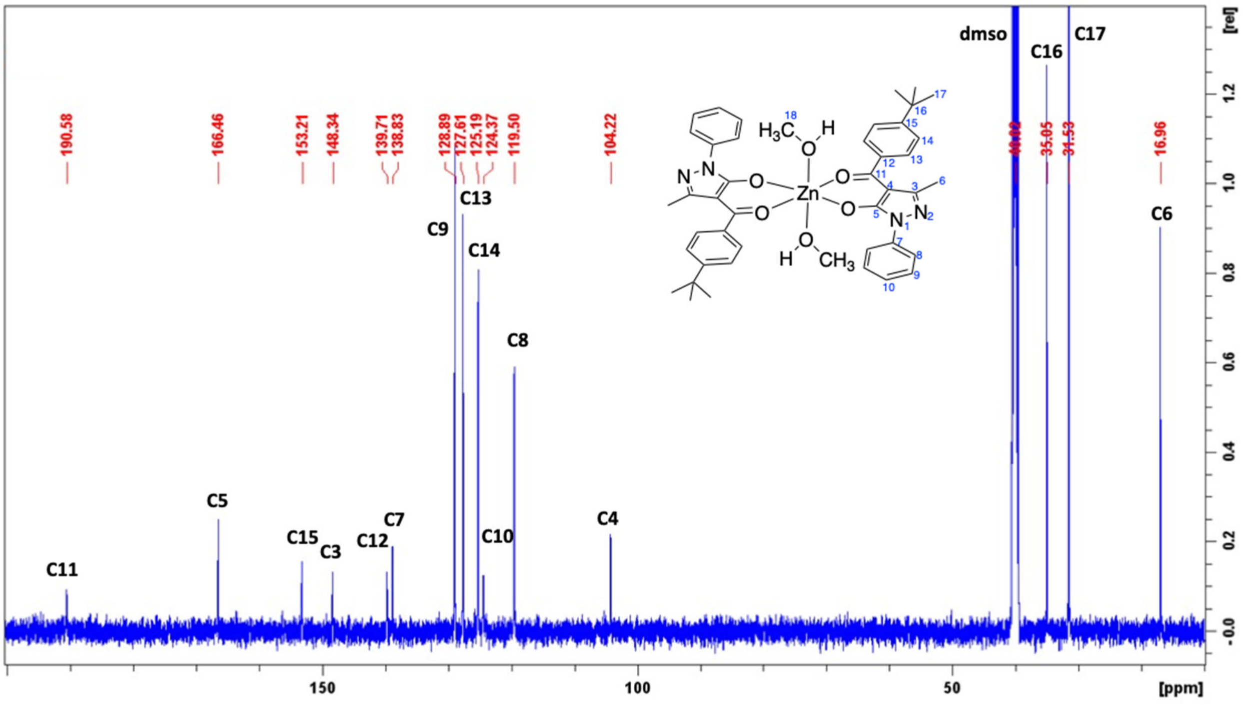

Figure S1. ^{1}H- NMR (**A**) and ^{13}C-NMR (**B**) of [Zn(Q^{ph})$_2$(MeOH)$_2$] complex with the molecular structure scheme.

Table S1 Details of data collection and structure refinements for [Zn(Q^{PhtBu})$_2$(MeOH)$_2$] complex

	**[Zn(Q^{PhtBu})$_2$(MeOH)$_2$]**
formula	C_{44} H_{50} N_4 O_6 Zn
Mr	796.25
crystal size [mm]	0.40 x 0.40 x 0.20
crystal system	Monoclinic
space group	*P2/c*
a [Å]	14.9509(9)
b [Å]	15.9318(9)
c [Å]	9.2965(6)
α [°]	90
β [°]	105.519(3)
γ [°]	90
V [Å^3]	2133.6(2)
Z	2
ρ cald [g cm^{-3}]	1.239
μ [mm^{-1}]	0.625
θ range [°]	2.557 to 26.374
data collected	38563
unique data, R_{int}	4327, 0.0401
obs. data [$I > 2\sigma(I)$]	4327
no. Parameters	291
restraints	32
R_1 [obs. data]	0.0387
wR_2 [all data]	0.1156
GOF	1.013

Table S2 Relevant bond distances (Å) and angles (°) for [Zn(Q^{PhtBu})$_2$(MeOH)$_2$] complex

Zn(1)-O(2)	2.0595(13)
Zn(1)-O(1)	2.0980(15)
Zn(1)-O(3)	2.1431(16)
O(2)-Zn(1)-O(1)	89.50(6)
O(1)-Zn(1)-O(3)	92.88(7)
O(2)-Zn(1)-O(3)	88.55(6)

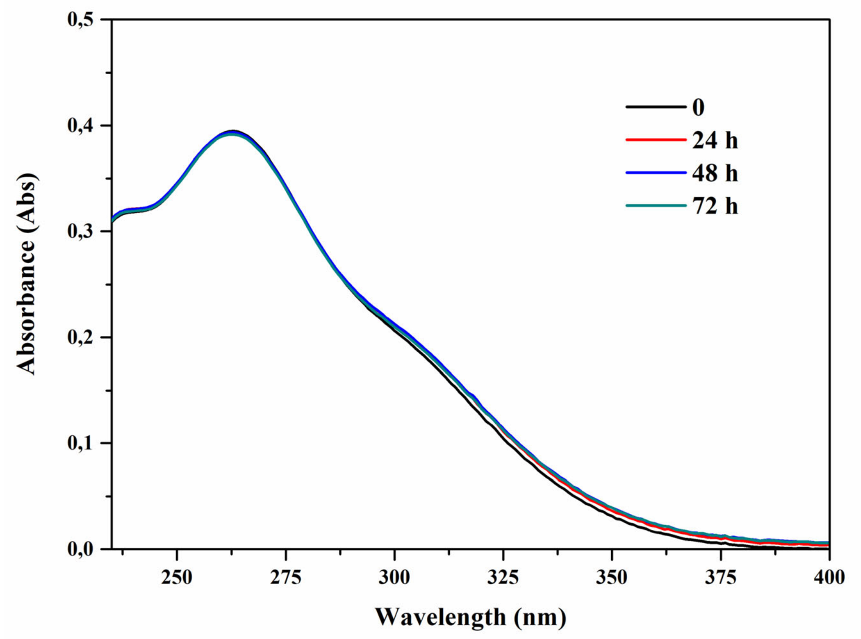

Figure S2. Absorption spectra recorded at 0, 24, 48 and 72 h of the $[Zn(Q^{PhtBu})_2(MeOH)_2]$ complex dissolved in the same mixture (acetic acid/methanol) used for the preparation of the CS@Zn$_n$ films.

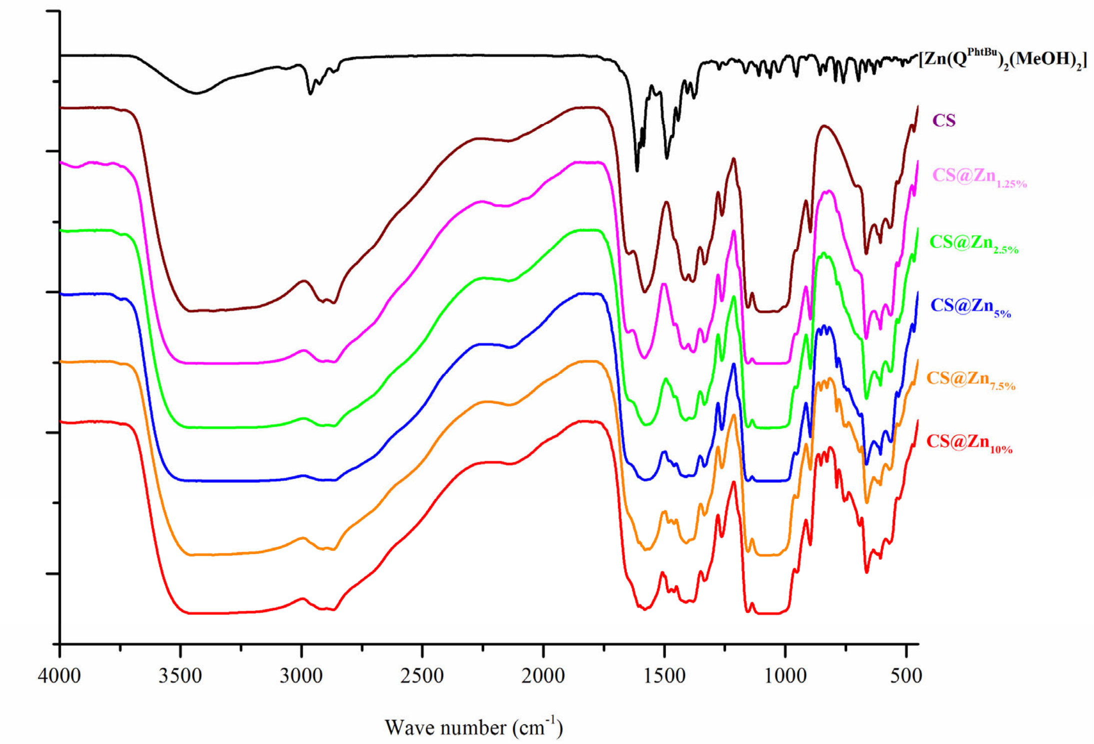

Figure S3. FT-IR spectra of all the CS@Zn$_n$ films and $[Zn(Q^{PhtBu})_2(MeOH)_2]$ complex.

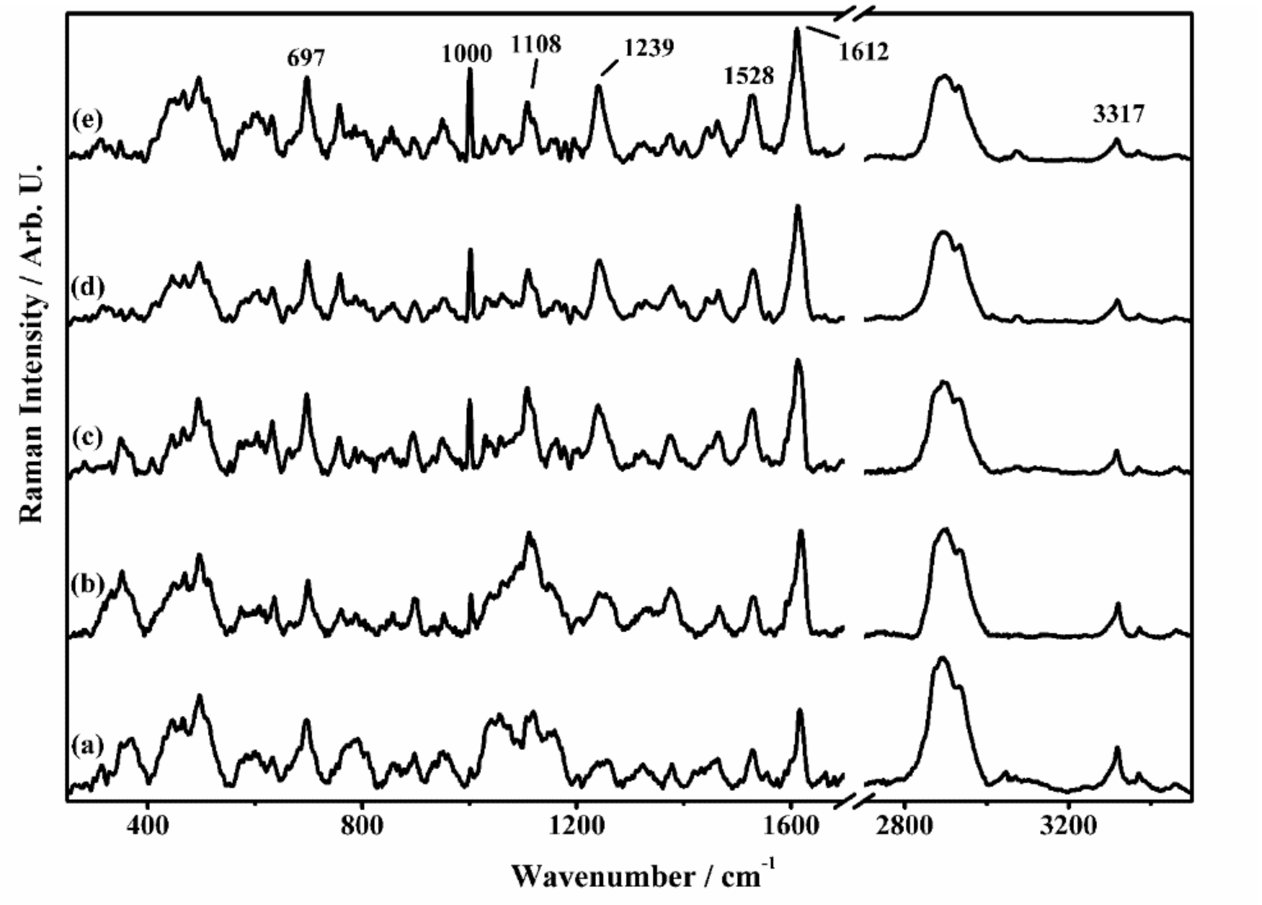

Figure S4. Representative Raman spectra in the ranges between 250 cm^{-1} and 1700 cm^{-1} and between 2700 cm^{-1} and 3500 cm^{-1} of CS@Zn_n films.

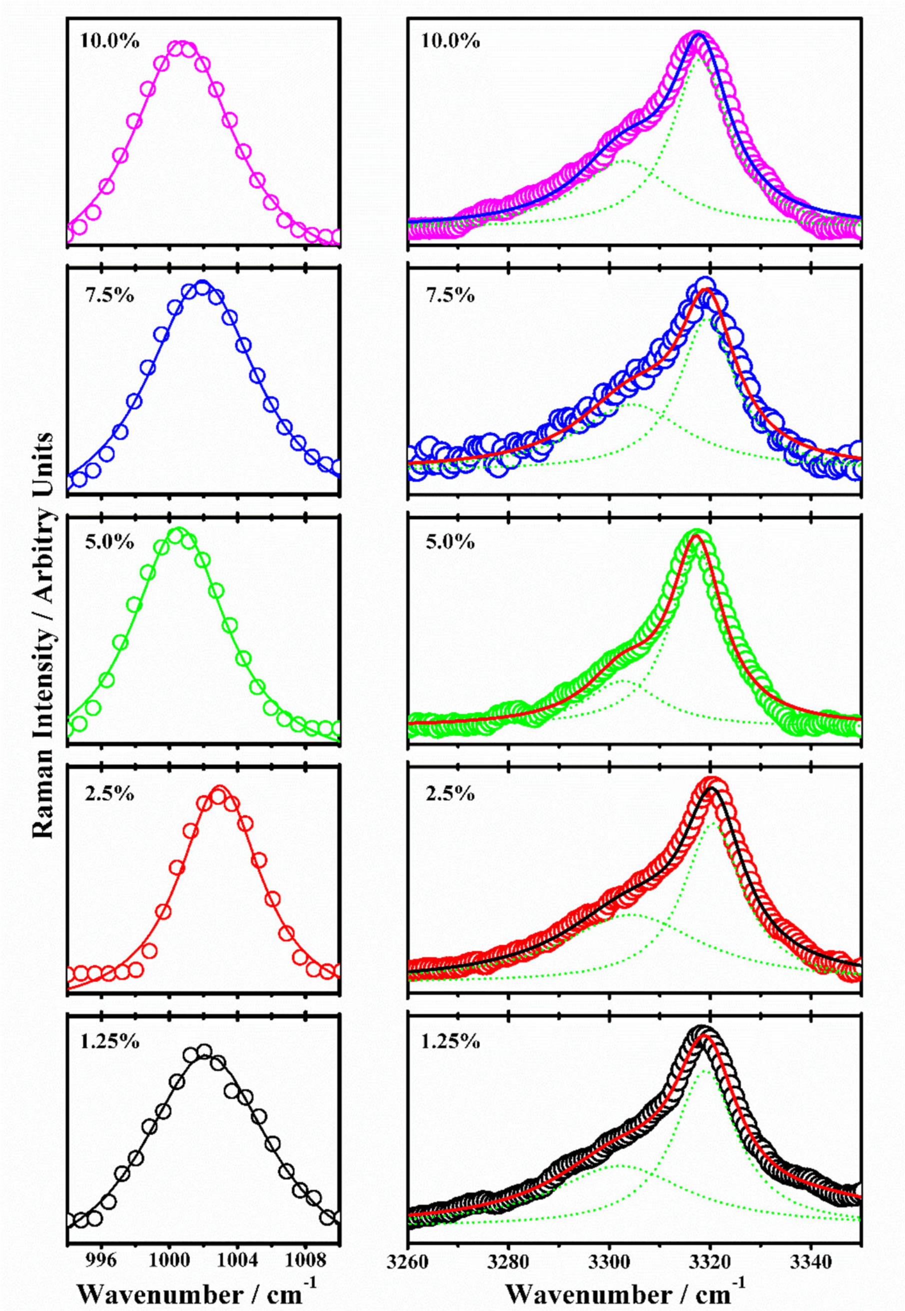

Figure S5. Representative Raman spectra in the range between 994 cm^{-1} and 1010 cm^{-1} and between 3260 cm^{-1} and 3350 cm^{-1} of the CS@Zn_n films. The open circle are the experimental data, the short dot curves are the single Lorentzian bands and the solid line is the total fitting curves.

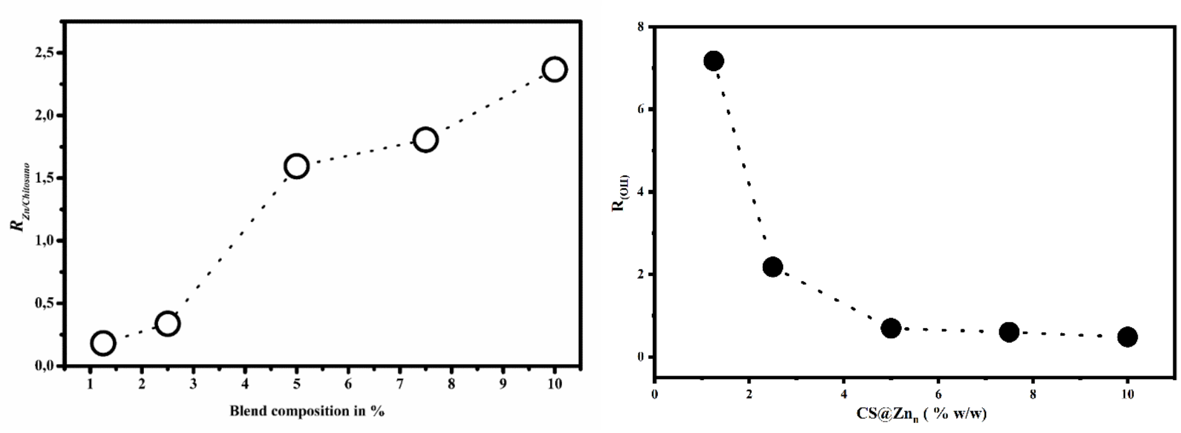

Figure S6. Ratio of the Intensity of the [Zn(Q^{PhtBu})$_2$(MeOH)$_2$] complex band at 1000 cm^{-1} and the sum of the bands at 3000 cm^{-1} and 3200 cm^{-1} (**A**) and the sum of the bands at 3371 cm^{-1} and 3457 cm^{-1} and the intensity of the [Zn(Q^{PhtBu})$_2$(MeOH)$_2$] complex band at 1000 cm^{-1} (**B**) as a function of the [Zn(Q^{PhtBu})$_2$(MeOH)$_2$] complex content in the CS film, in percentage.

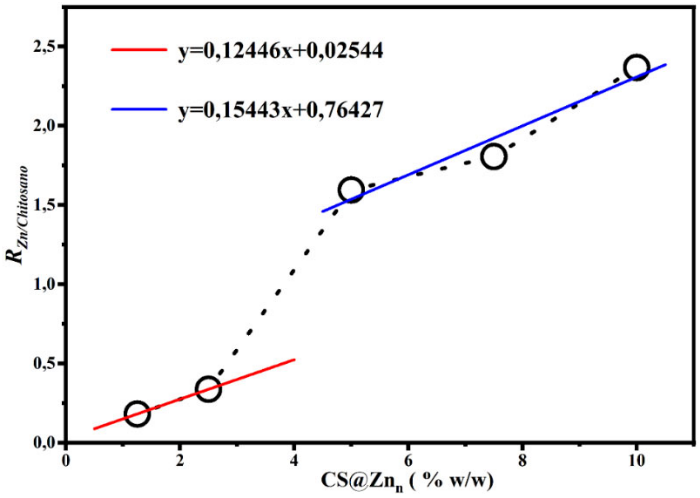

Figure S7. Intensity Ratio of the Intensity of the Zn complex band at 1000 cm^{-1} and the sum of the bands at 3300 cm^{-1} and 3320 cm^{-1} as a function of the film composition in percentage. The red line represents the linear fit performed on the first two points whereas the blue line represents the linear fit obtained for the last three points

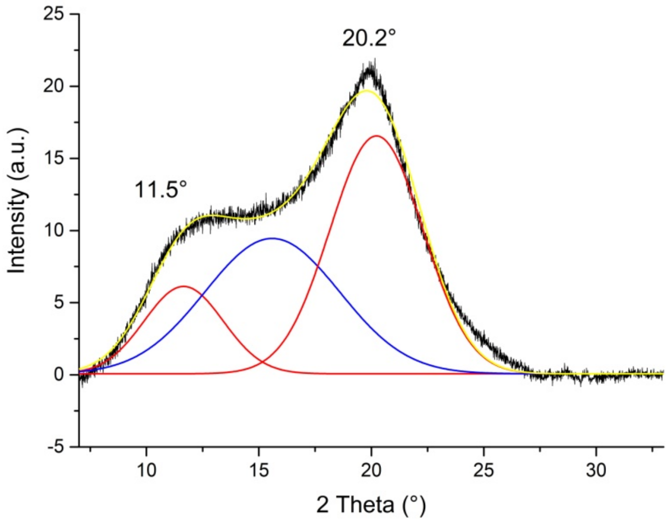

Figure S8. XRD profile of the CS powder sample with its deconvolution curves (crystalline peaks: red lines; amorphous region: blue curve; cumulative fit curve: yellow line).

a)

b)

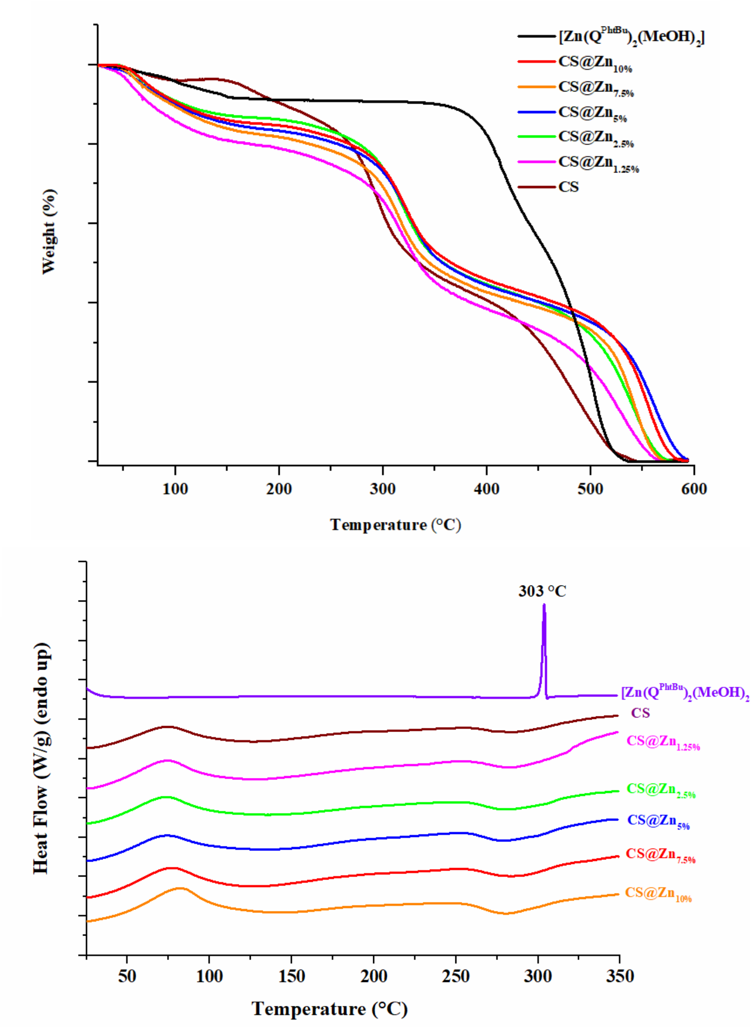

Figure S9. (**A**) TGA curves, (**B**) DSC traces of [Zn(Q^{PhtBu})$_2$(MeOH)$_2$], CS, CS@Zn$_{1.25\%}$, CS@Zn$_{2.5\%}$, CS@Zn$_{5\%}$, CS@Zn$_{7.5\%}$ and CS@Zn$_{10\%}$ films.

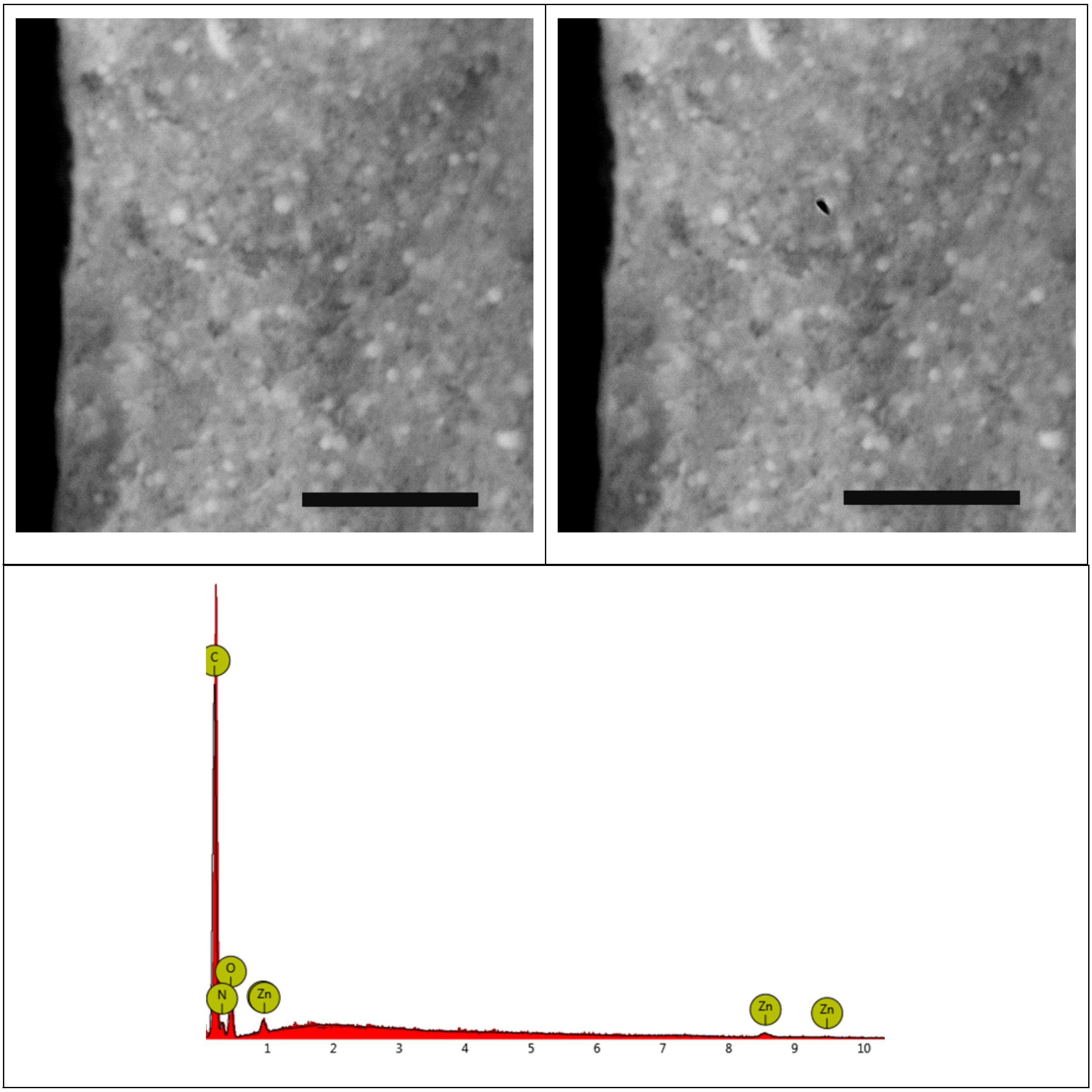

Figure S10. Detail of observed amorphous aggregates present in CS@Zn_n films, before (left) and after (right) punctual EDX analyses (Scale Bar: 5 μm). In inset, EDX pattern of punctual analysis. Note that owe to the occurring thermal decomposition, analyses were rapidly stopped to preserve the integrity of the instrument, consequently EDX pattern can only be considered of an indicative nature rather than accurate quantitative analysis

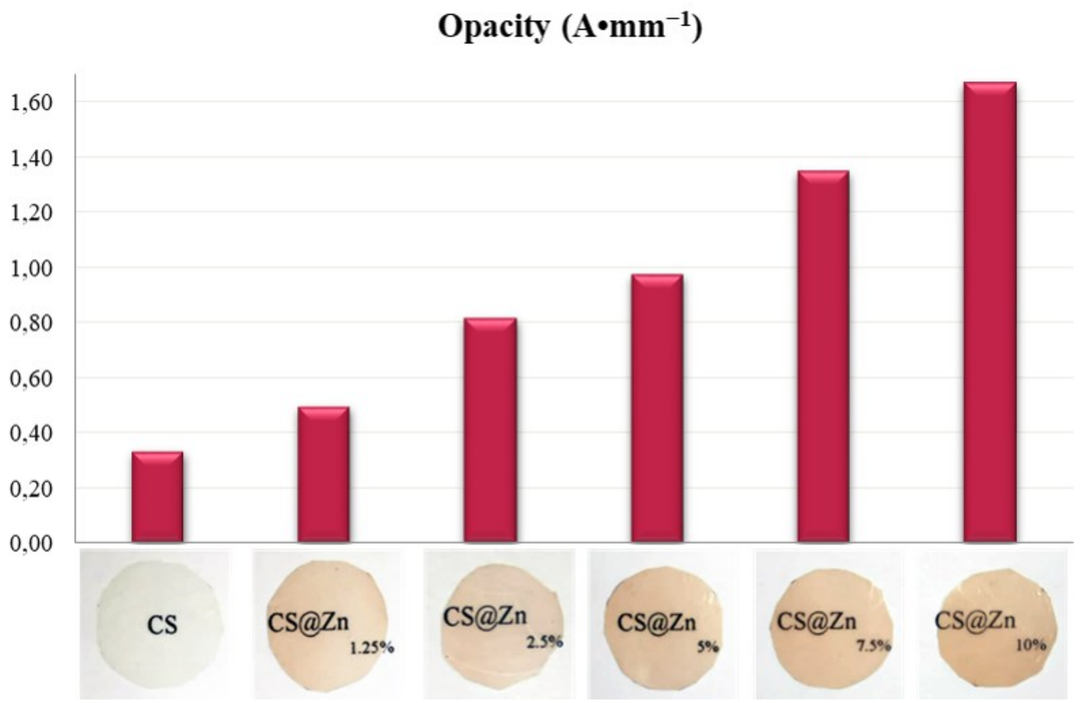

Figure S11. Opacity measurements of the prepared films together with photographs of the films placed over written captions to show their overall color and transparency.

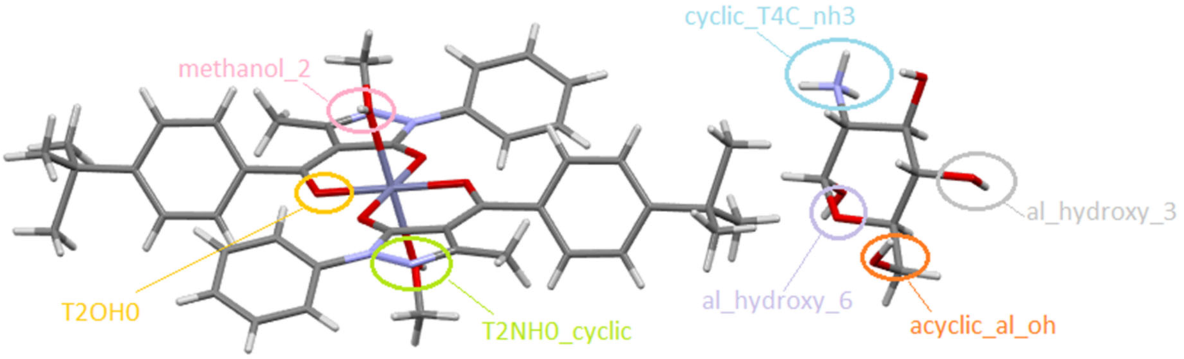

Figure S12. Hydrogen bonding groups present within the target paired-molecules

Table S3. Hydrogen bond propensity calculation for the studied model

Donor	Acceptor	Propensity (lower and upper bound)	Frequency
methanol_2	al_hydroxy_3	0.92 (0.84-0.96)	0.5
methanol_2	T2NH0_cyclic	0.89 (0.80-0.94)	1.8
methanol_2	acyclic_al_oh	0.86 (0.76-0.93)	0.1
cyclic_T4C_nh3	T2NH0_cyclic	0.86 (0.83-0.89)	71.4

a)

b)

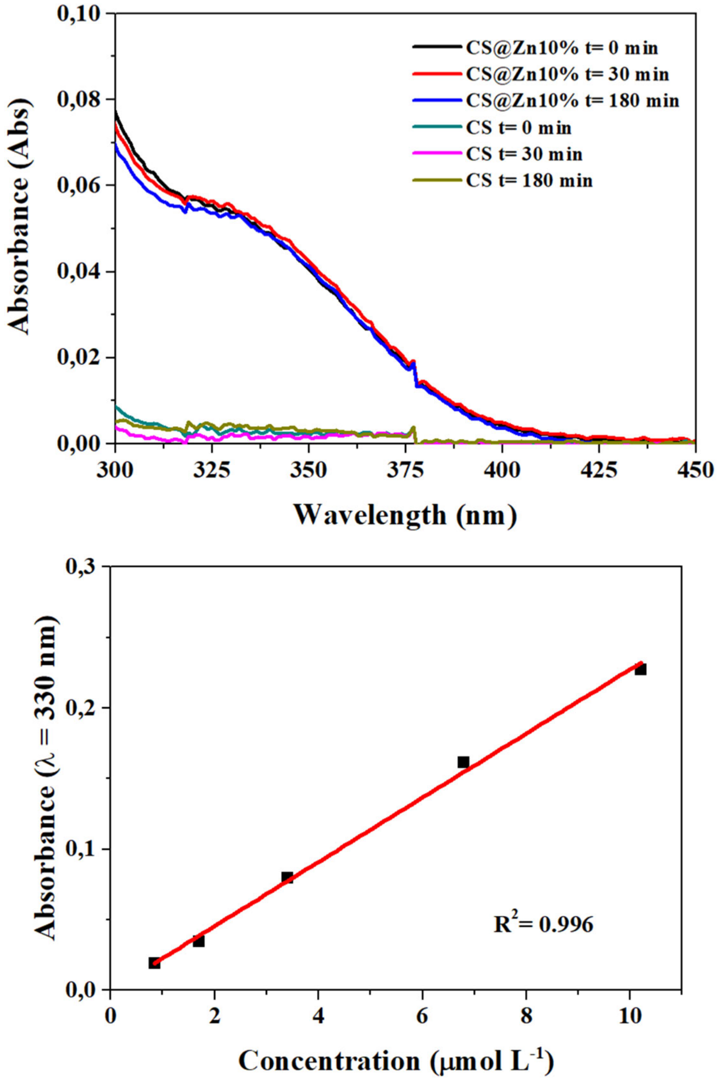

Figure S13. (**A**) Representative spectra of the PBS solution obtained after different times of immersion of $CS@Zn_{10\%}$ film and CS film (control) (t = 0 min, t = 30 min, t = 180 min). (**B**) Calibration curve of $[Zn(Q^{PhtBu})_2(MeOH)_2]$ in methanol then diluted in PBS (methanol 4% v/v).

a)

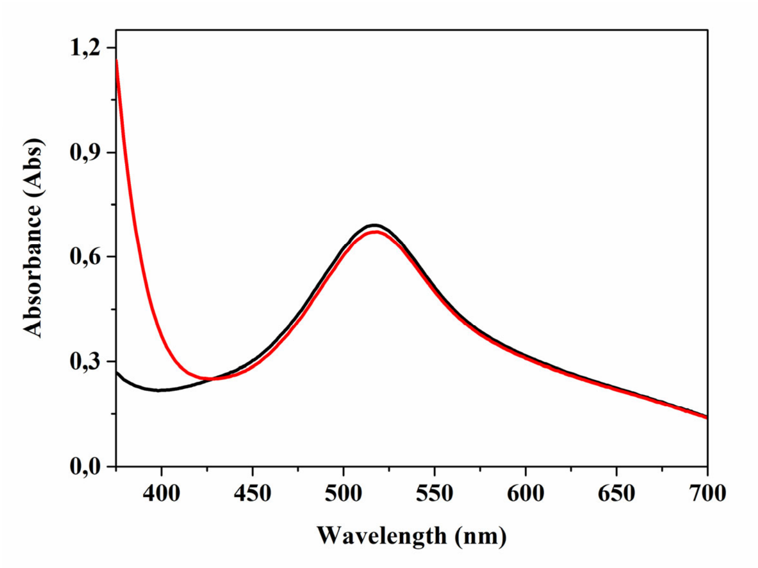

b)

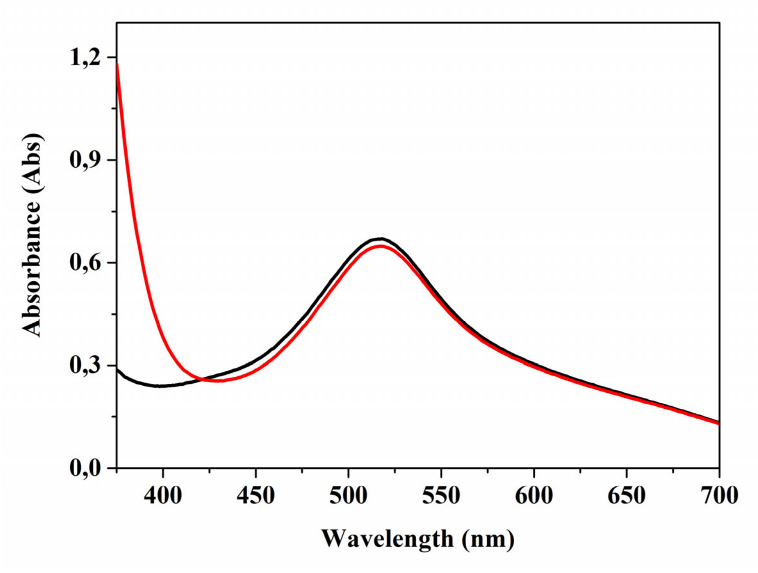

Figure S14. Absorption spectra of DPPH ethanolic solution (black) and after incubation for 3 (**A**) and 24 h (**B**) with $[Zn(Q^{PhtBu})_2(MeOH)_2]$ (red).

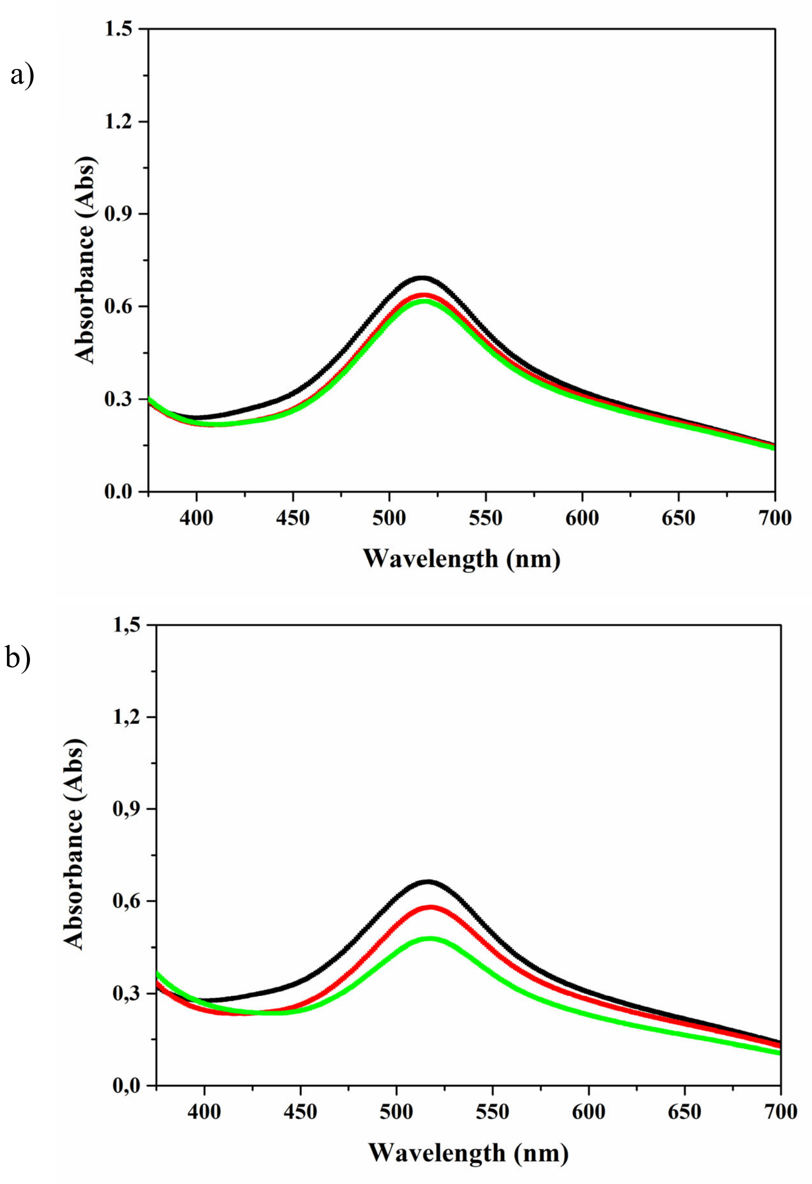

Figure S15. Absorption spectra of DPPH ethanolic solution (black) and after incubation for 3 (**A**) and 24 h (**B**) with chitosan films CS@AA$_{1.25\%}$ (red) and CS@AA$_{10\%}$ (green), incorporating $2.89 \cdot 10^{-6}$ and $2.29 \cdot 10^{-5}$ mol of ascorbic acid, respectively.

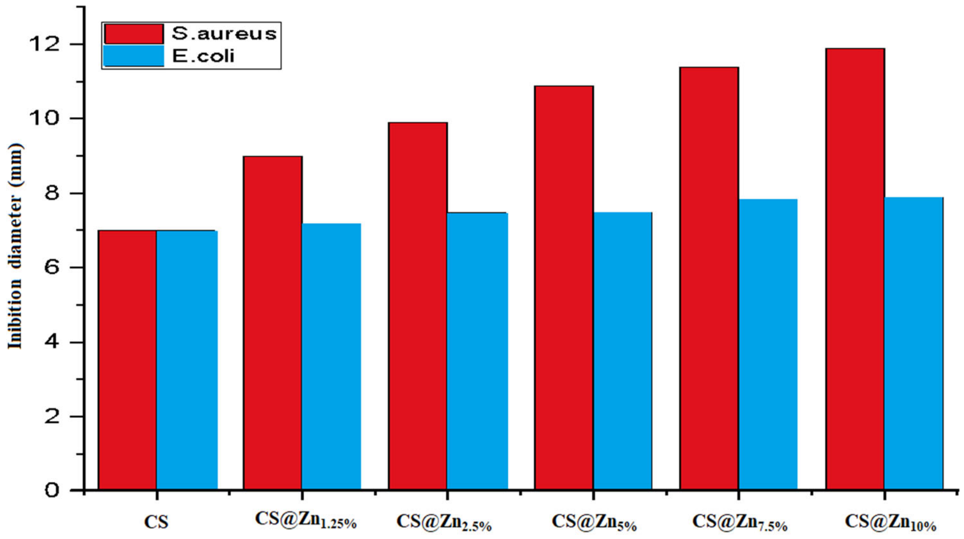

Figure S16. In vitro antimicrobial activity of chitosan membranes and chitosan/zinc complex membranes against *S.aureus* and *E.coli*.
