# Supplementary material for: New Zinc-Based Active Chitosan Films: Physicochemical Characterization, Antioxidant, and Antimicrobial Properties
Source: Front Chem. 2022 May 31;10:884059. doi: 10.3389/fchem.2022.884059 (PMC9194505; doi:10.3389/fchem.2022.884059)

checkCIF/PLATON report

Structure factors have been supplied for datablock(s) shelx_zinc_complex

THIS REPORT IS FOR GUIDANCE ONLY. IF USED AS PART OF A REVIEW PROCEDURE FOR PUBLICATION, IT SHOULD NOT REPLACE THE EXPERTISE OF AN EXPERIENCED CRYSTALLOGRAPHIC REFEREE.

No syntax errors found. CIF dictionary Interpreting this report

Datablock: shelx_zinc_complex

Bond precision:	C-C = 0.0042 A		Wavelength=0.71073
Cell:	a=14.9509(9)	b=15.9318(9)	c=9.2965(6)
	alpha=90	beta=105.519(3)	gamma=90
Temperature:	296 K		

	Calculated	Reported
Volume	2133.7(2)	2133.6(2)
Space group	P 21/c	P 21/c
Hall group	-P 2ybc	-P 2ybc
Moiety formula	C43.29 H47.87 N4 O6 Zn, 0.71(C H3)	C43.29 H47.87 N4 O6 Zn, 0.71(C H3)
Sum formula	C44 H50 N4 O6 Zn	C44 H50 N4 O6 Zn
Mr	796.27	796.27
Dx,g cm-3	1.239	1.239
Z	2	2
Mu (mm-1)	0.625	0.625
F000	840.0	840.0
F000'	840.89	
h,k,lmax	18,19,11	18,19,11
Nref	4362	4327
Tmin,Tmax	0.787,0.882	0.796,0.878
Tmin'	0.771	

Correction method= # Reported T Limits: Tmin=0.796 Tmax=0.878
AbsCorr = EMPIRICAL

Data completeness= 0.992 Theta(max)= 26.374

R(reflections)= 0.0387(3290) wR2(reflections)= 0.1156(4327)

S = 1.013 Npar= 291

The following ALERTS were generated. Each ALERT has the format
test-name_ALERT_alert-type_alert-level.
Click on the hyperlinks for more details of the test.

Alert level C

```
PLAT220_ALERT_2_C NonSolvent   Resd 1  C   Ueq(max)/Ueq(min) Range        4.9 Ratio
PLAT222_ALERT_3_C NonSolvent Resd 1  H   Uiso(max)/Uiso(min) Range        4.7 Ratio
PLAT230_ALERT_2_C Hirshfeld Test Diff for    C14      --C15      .        6.3 s.u.
PLAT242_ALERT_2_C Low    'MainMol' Ueq as Compared to Neighbors of        C15 Check
PLAT260_ALERT_2_C Large Average Ueq of Residue Including      C21A      0.126 Check
PLAT911_ALERT_3_C Missing FCF Refl Between Thmin & STh/L=    0.600          2 Report
```

Alert level G

```
PLAT002_ALERT_2_G Number of Distance or Angle Restraints on AtSite          7 Note
PLAT003_ALERT_2_G Number of Uiso or Uij Restrained non-H Atoms ...          4 Report
PLAT007_ALERT_5_G Number of Unrefined Donor-H Atoms .............           1 Report
PLAT176_ALERT_4_G The CIF-Embedded .res File Contains SADI Records          2 Report
PLAT177_ALERT_4_G The CIF-Embedded .res File Contains DELU Records          2 Report
PLAT230_ALERT_2_G Hirshfeld Test Diff for    C18      --C20      .       19.3 s.u.
PLAT300_ALERT_4_G Atom Site Occupancy of C22        Constrained at        0.6 Check
PLAT300_ALERT_4_G Atom Site Occupancy of C22A       Constrained at        0.4 Check
PLAT300_ALERT_4_G Atom Site Occupancy of H22A       Constrained at        0.6 Check
PLAT300_ALERT_4_G Atom Site Occupancy of H22B       Constrained at        0.6 Check
PLAT300_ALERT_4_G Atom Site Occupancy of H22C       Constrained at        0.6 Check
PLAT300_ALERT_4_G Atom Site Occupancy of H22D       Constrained at        0.4 Check
PLAT300_ALERT_4_G Atom Site Occupancy of H22E       Constrained at        0.4 Check
PLAT300_ALERT_4_G Atom Site Occupancy of H22F       Constrained at        0.4 Check
PLAT301_ALERT_3_G Main Residue  Disorder .............(Resd  1  )         13% Note
PLAT302_ALERT_4_G Anion/Solvent/Minor-Residue Disorder (Resd  2  )       100% Note
PLAT304_ALERT_4_G Non-Integer Number of Atoms in ..... (Resd  1  )     102.16 Check
PLAT304_ALERT_4_G Non-Integer Number of Atoms in ..... (Resd  2  )       1.42 Check
PLAT367_ALERT_2_G Long?  C(sp?)-C(sp?) Bond  C15      - C18      .       1.52 Ang.
PLAT413_ALERT_2_G Short Inter XH3 .. XHn     H14      ..H21E     .       2.04 Ang.
                                                      x,y,z  =      1_555 Check
PLAT413_ALERT_2_G Short Inter XH3 .. XHn     H14      ..H21F     .       2.09 Ang.
                                                      x,y,z  =      1_555 Check
PLAT414_ALERT_2_G Short Intra D-H..H-X       H3A      ..H22C     .       2.12 Ang.
                                                      x,y,z  =      1_555 Check
PLAT414_ALERT_2_G Short Intra D-H..H-X       H3A      ..H22D     .       2.09 Ang.
                                                      x,y,z  =      1_555 Check
PLAT432_ALERT_2_G Short Inter X...Y Contact  C14      ..C21A     .       2.78 Ang.
                                                      x,y,z  =      1_555 Check
PLAT432_ALERT_2_G Short Inter X...Y Contact  C15      ..C21A     .       2.65 Ang.
                                                      x,y,z  =      1_555 Check
PLAT432_ALERT_2_G Short Inter X...Y Contact  C18      ..C21A     .       1.78 Ang.
                                                      x,y,z  =      1_555 Check
PLAT764_ALERT_4_G Overcomplete CIF Bond List Detected (Rep/Expd) .       1.11 Ratio
PLAT773_ALERT_2_G Check long C-C Bond in CIF: C18       --C20            1.73 Ang.
PLAT773_ALERT_2_G Check long C-C Bond in CIF: C18       --C21A           1.78 Ang.
PLAT779_ALERT_4_G Suspect or Irrelevant (Bond) Angle(s) in CIF ...       0.00 Deg.
              O2   -O2   -C3      1_555   1_555   1_555 ......     #   22 Check
PLAT779_ALERT_4_G Suspect or Irrelevant (Bond) Angle(s) in CIF ...       0.00 Deg.
              O2   -O2   -ZN1     1_555   1_555   1_555 ......     #   23 Check
PLAT779_ALERT_4_G Suspect or Irrelevant (Bond) Angle(s) in CIF ...       0.00 Deg.
```

```
              O2    -C3    -O2      1_555   1_555   1_555 ......     #   26 Check
PLAT802_ALERT_4_G CIF Input Record(s) with more than 80 Characters          1 Info
PLAT860_ALERT_3_G Number of Least-Squares Restraints ............          32 Note
PLAT883_ALERT_1_G No Info/Value for _atom_sites_solution_primary .     Please Do !
PLAT910_ALERT_3_G Missing # of FCF Reflection(s) Below Theta(Min).          2 Note
PLAT912_ALERT_4_G Missing # of FCF Reflections Above STh/L=  0.600         31 Note
PLAT933_ALERT_2_G Number of HKL-OMIT Records in Embedded .res File          3 Note
PLAT965_ALERT_2_G The SHELXL WEIGHT Optimisation has not Converged     Please Check
PLAT978_ALERT_2_G Number C-C Bonds with Positive Residual Density.          3 Info
```

```
   0 ALERT level A = Most likely a serious problem - resolve or explain
   0 ALERT level B = A potentially serious problem, consider carefully
   6 ALERT level C = Check. Ensure it is not caused by an omission or oversight
  40 ALERT level G = General information/check it is not something unexpected

   1 ALERT type 1 CIF construction/syntax error, inconsistent or missing data
  20 ALERT type 2 Indicator that the structure model may be wrong or deficient
   5 ALERT type 3 Indicator that the structure quality may be low
  19 ALERT type 4 Improvement, methodology, query or suggestion
   1 ALERT type 5 Informative message, check
```

It is advisable to attempt to resolve as many as possible of the alerts in all categories. Often the minor alerts point to easily fixed oversights, errors and omissions in your CIF or refinement strategy, so attention to these fine details can be worthwhile. In order to resolve some of the more serious problems it may be necessary to carry out additional measurements or structure refinements. However, the purpose of your study may justify the reported deviations and the more serious of these should normally be commented upon in the discussion or experimental section of a paper or in the "special_details" fields of the CIF. checkCIF was carefully designed to identify outliers and unusual parameters, but every test has its limitations and alerts that are not important in a particular case may appear. Conversely, the absence of alerts does not guarantee there are no aspects of the results needing attention. It is up to the individual to critically assess their own results and, if necessary, seek expert advice.

Publication of your CIF in IUCr journals

A basic structural check has been run on your CIF. These basic checks will be run on all CIFs submitted for publication in IUCr journals (*Acta Crystallographica*, *Journal of Applied Crystallography*, *Journal of Synchrotron Radiation*); however, if you intend to submit to *Acta Crystallographica Section C* or *E* or *IUCrData*, you should make sure that full publication checks are run on the final version of your CIF prior to submission.

Publication of your CIF in other journals

Please refer to the *Notes for Authors* of the relevant journal for any special instructions relating to CIF submission.

PLATON version of 19/02/2022; check.def file version of 19/02/2022

Datablock shelx_zinc_complex - ellipsoid plot

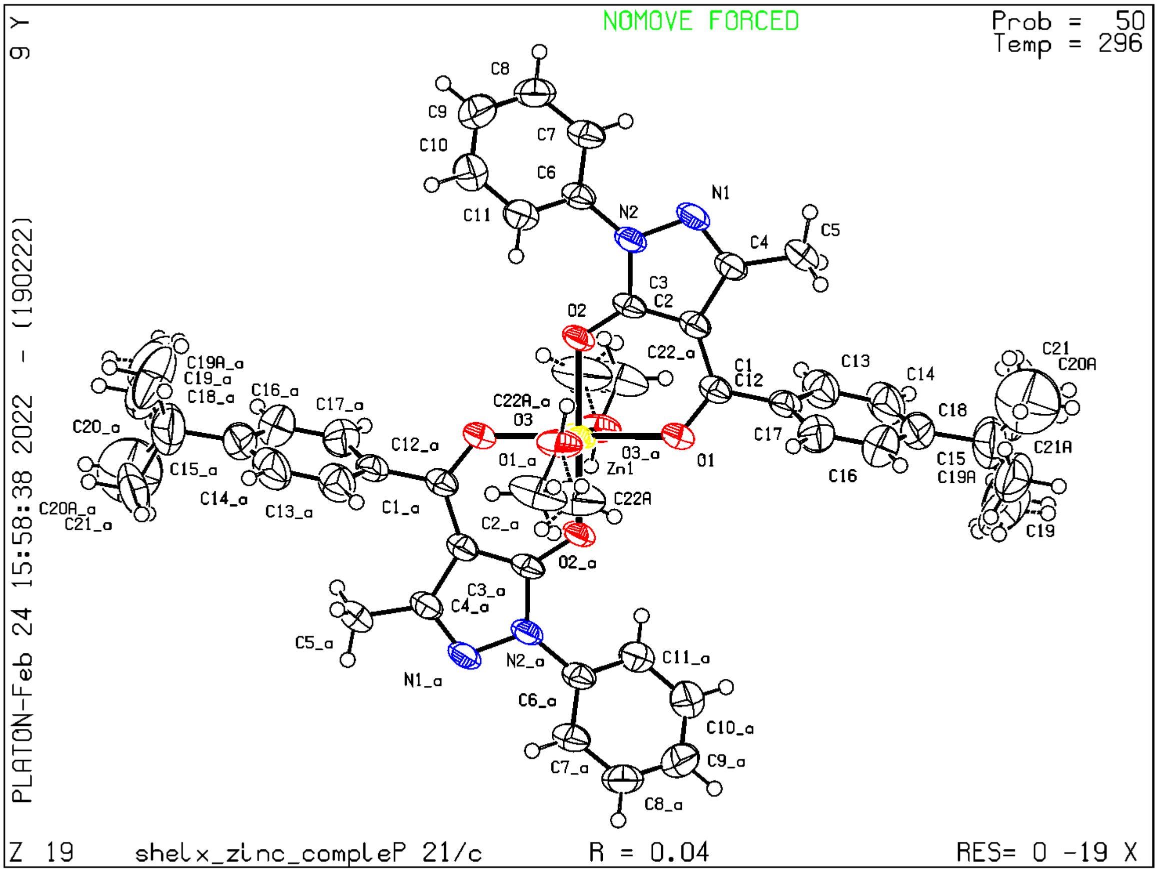

Supplement: Supplementary file 2 [file DataSheet1.PDF]
